# Computational Analysis of a Next-Generation Platinum-Based Chemotherapies that Induce DNA Double-Strand Breaks

**DOI:** 10.1021/acs.jcim.5c01654

**Published:** 2025-11-22

**Authors:** Amanda R. Guimarães, Óscar R. Ballesteros, Iván Rivilla, Irene Olaizola, Mikel Odriozola-Gimeno, Abel de Cózar, David de Sancho, Xabier Lopez, Jesus M. Banales, Fernando P. Cossío

**Affiliations:** 1 Department of Organic Chemistry I, Center of Innovation in Advanced Chemistry (ORFEO−CINQA), Faculty of Chemistry, University of the Basque Country (UPV/EHU), P° Manuel Lardizabal 3, Donostia/San Sebastián 20018, Spain; 2 Donostia International Physics Center (DIPC), P° Manuel Lardizabal 4, Donostia/San Sebastian 20018, Spain; 3 IKERBASQUE, Basque Foundation for Science, M^a^ Diaz de Haro 3, Bilbao 48013, Spain; 4 Department of Liver and Gastrointestinal Diseases, Biogipuzkoa Health Research Institute - Donostia University Hospital -, University of the Basque Country (UPV/EHU), P° Dr. Begiristain, s/n, Donostia-San Sebastian 20014, Spain; 5 Polimero eta Material Aurreratuak: Fisika, Kimika eta Teknologia & Donostia International Physics Center (DIPC), P° Manuel Lardizabal 3, Donostia/San Sebastian 20018, Spain; 6 National Institute for the Study of Liver and Gastrointestinal Diseases (CIBERehd, ″Instituto de Salud Carlos III″) &Department of Biochemistry and Genetics, School of Sciences, University of Navarra, C/Irunlarrea, 1, Pamplona 31008, Navarra, Spain

## Abstract

Platinum-based chemotherapeutic agents, such as cisplatin (Cis-Pt­(II)), are widely used in cancer treatment but are limited by toxicity, DNA repair by cancer cells, and drug resistance. To address these limitations, we designed and synthesized in our laboratories a novel platinum-based compound, Aurkine16. In our previous studies, Aurkine16 demonstrated outstanding therapeutic efficacy, selectively inducing double-strand DNA breaks in both naïve and cisplatin-resistant cancer cells, without detectable toxic side effects at clinically relevant doses. In the present work, we report a computational analysis of Aurkine16, which reveals its unique activation pathway and its capacity to form stable [Aurki-GGG]^3+^ complexes. Unlike Cis-Pt­(II), which induces single-strand DNA breaks, Aurkine16 simultaneously targets multiple nucleic bases, causing double-strand breaks and significant DNA disruption. Additionally, molecular dynamics simulations suggest that Aurkine16 is likely to exhibit specificity for cancer cells, avoiding off-target interactions within the nucleosome core. This selectivity, attributed to the steric hindrance from histone tails, underscores its potential for effectively targeting chromatin-accessible cancer cells. These computational findings position Aurkine16 as a promising alternative to platinum-based drugs, particularly for CisPt-resistant cancers.

## Graphical abstract

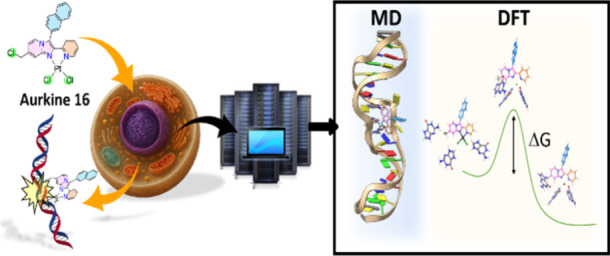

## Introduction

DNA-damaging agents remain crucial chemotherapies for treating cancer.^1^ Since the approval of cisplatin (Cis-Pt­(II)) in the 1970s, platinum-based drugs have played a vital role in oncology. Cisplatin, carboplatin, and oxaliplatin are the three platinum drugs approved for global clinical use. Although these agents share similar pharmacological properties, they differ in their anticancer activity and side effect profiles.^2^

Cis-Pt­(II) has been widely used to treat various cancers, including bladder, ovarian, head and neck, lung, testicular, cervical esophageal, breast, and biliary cancers.^3^ However, cisplatin and its analogues are associated with significant toxicity and off-target effects, such as nephrotoxicity, hematotoxicity, ototoxicity, neurotoxicity, and gastrointestinal disturbances, among others.^4^ Despite their clinical success, a high proportion of tumors treated with current platinum derivatives develop resistance and continue to progress, highlighting the urgent need for improved platinum-based therapies. Several strategies are being explored to address these challenges, including combination treatments,^5^ use of prodrugs^6^ (for instance, Pt (IV) compounds^7^ that are reduced to Pt (II) complexes *in vivo*), advanced drug delivery systems,^8^ and comprehensive exploration of the chemical space,^9^ among other strategies.

Typically, platinum-based drugs possess two leaving groups that enable DNA cross-linking through S_N_2 reactions, forming adducts such such as (1,2)-intrastrand, (1,3)-intrastrand, or (1,2’)-interstrand (Figure 1A). In these reactions, purine bases in DNA—primarily guanine (G) and adenine (A)— act as nucleophiles (:Nu), attacking the electrophilic platinum center (E) and displacing a leaving group (Lg), such as chloride or carboxylate (e.g., Cl^–^, RCO_2_^–^). In all types of resulting DNA-ligand complexes, the Pt–N bonds involve the N7 atom of purine bases.

**Figure 1:**
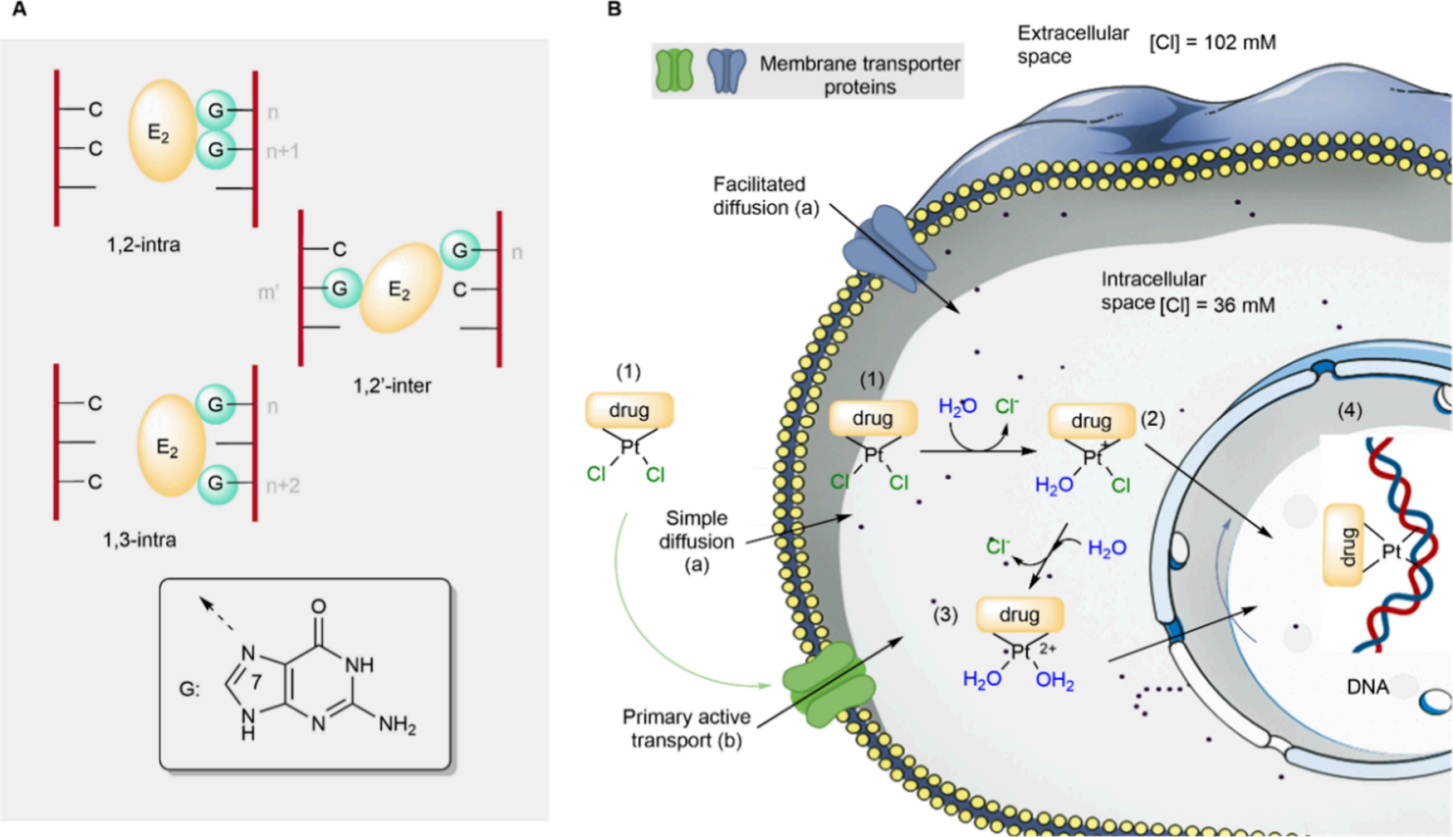
A, Dielectrophilic (E2) interaction with guanines (G). B, Reaction mechanism of platinum-based drug. The drugs can cross the cell and nuclear membrane through passive diffusion (a) or active transport (b).

The reaction mechanism of platinum-based drugs with their targets is not yet fully understood. However, it is hypothesized that these compounds initially exist in an approximately 50:50 neutral: aqua-cationic ratio in the extracellular medium, due to the high concentration of chloride ions. Upon entering the cytoplasm —where chloride levels are significantly lower—the drug becomes activated by surrounding water molecules, forming mono- or diaqua species (structures 2 and 3 in Figure 1B) through one or two S_N_2 reactions. In this process, water acts as the nucleophile (:Nu) while chloride, alkoxide, or carboxylate anions serve as leaving groups (Lg). These aquated intermediates then undergo further S_N_2 substitution with purine bases in DNA—primarily guanine—where the aqua ligands are replaced, forming covalent platinum-DNA adducts. This reaction results in considerable distortion of the DNA double helix (structure 4 in Figure 1B), triggering a cellular damage response that can ultimately lead to cancer cell death.^10–13^ However, the DNA lesions caused by these drugs can often be efficiently repaired by cellular DNA repair mechanisms, thereby limiting the overall therapeutic efficacy, particularly during prolonged or repeated treatment.^14^

Those repair mechanisms are particularly effective against (1,2)- and (1,3)-intrastrand cross-links, which often result in single-strand DNA breaks—the most common type of lesions.^15^ In contrast, interstrand cross-links—which cause double-strand DNA breaks—are significantly more difficult for cancer cells to repair.^16,17^ However, such interstrand cross-links are relatively rare, occurring in fewer than 5% of cases.^15,18^

To overcome this limitation, a new class of platinum-based compounds known as Aurkines was developed.^19^ These molecules were designed to increase the number of electrophilic positions (En), with the aim of enhancing the formation of interstrand cross-link adducts that would result in irreversible DNA damage in cancer cells.

To achieve this, several innovative strategies were incorporated into the design of Aurkines. These included the incorporation of intercalating (π-stacking) moieties,^20^ the addiction of basic centers to improve transport by organic cationic transporters,^21^ and the development of fluorophores to facilitate tracking the distribution of molecules within cellular organelles.

In our previous study,^19^ Aurkines—particularly Aurkine16—were shown to effectively induce double-strand DNA breaks, leading to increased DNA damage and elevated levels of reactive oxygen species. This resulted in greater cytotoxicity compared to Cis-Pt­(II), specifically in human cholangiocarcinoma (CCA) cells *in vitro*, with no harmfull effects on normal cells. Moreover, Aurkine16 demonstrated efficacy against CCA cells resistant to Cis-Pt­(II), highlighting its potential as a promising candidate for cancer therapy. To understand how Aurkine16 overcomes Cis-Pt­(II) resistance in CCA cells, it is essential to investigate how this drug candidate alters the structure of its primary target, the DNA. In the case of Cis-Pt­(II), X-ray diffraction analysis of duplex DNA containing a Cis-Pt­(II) 1,2-d­(GpG) intrastrand cross-link revealed that the double helix bends 35–40° toward the major groove.^22^ However, the structure of the cross-links formed with DNA-Aurkines complexes remains unclear, likely due to the poor diffraction capabilities of these adducts. In this context, computational chemistry tools based on quantum mechanics and molecular modeling are essential for better understanding DNA damage and repair following the formation of these complexes, which disrupt the double helix. Specifically, we have employed computational chemistry methods utilizing Quantum Mechanics (QM), based on Density Functional Theory (DFT), and Molecular Dynamics (MD), including a Molecular Mechanics (MM) force field, to investigate how Aurkine16 compromises the structure of DNA sequences.

## Materials and Methods

### QM Calculations

The QM calculations of stationary points were performed within the DFT framework. The B3LYP^23^ exchange-correlation functional, combined with Grimme’s D3 ^24^ dispersion correction and Baker & Johnson (BJ)^25^ damping function, was used as it offers a good balance between accuracy and computational cost, while acknowledging its known constraints.^26,27^ Solvent effects were treated by including both explicit water molecules and the Polarizable Continuum Model^28^ (PCM) for water (ε = 78.3553), to account for nonspecific solute–solvent interactions through a continuum dielectric description. Up to four explicit water molecules were incorporated to assess the influence of microsolvation on the system. Harmonic vibrational analyses were performed to characterize the stationary points. Reactants and products exhibited positive definite Hessians, while transition states were identified by the presence of a single imaginary frequency. The absolute energies of all stationary points discussed in this study, along with the corresponding imaginary frequency values, are provided in the Supporting Information (Tables S1, S3, S5, S7, S9, and S10). Thermochemical properties, including Gibbs free energies, enthalpies, and zero-point energies, were calculated at a pressure of 1 atm and a temperature of 298.15 K. All DFT calculations were run using the Gaussian16 ^29^ computational package.

### Parameter Generation

Parameter generation for Aurkine16 was described preveously.^19^ For Cis-Pt­(II), and constructs involving ligands bonded to guanine bases, parameters were generated using the MCPB.py script,^30^ following the Amber tutorial “Building Bonded Model for a Ligand Binding Metalloprotein with MCPB.py”. Briefly, this approach combines the General Amber Force Field (GAFF)^31^ with structure and charge optimization at the quantum level. Coordinate files for the ligand, metal ion, and guanine bases bonded to the ligands were generated,^32^ followed by structure optimization, force constants determination, and Merz–Kollman RESP^33^ charges calculations using Gaussian16 software,^29^ with the B3LYP^23^/def2-SVP^34^ model chemistry. When ligands were bound to one or more guanine bases, nucleotides lacking the sugar–phosphate backbone were included to allow greater flexibility during calculations. The resulting parameters and partial charges were incorporated into the system force fields and can be found in the Zenodo Repository.

### DNA Double-Strand Molecular Dynamics Simulations

System setup was performed using LEaP software within the AmberTools^35^ suite.

The DNA sequence d­(GCACGAACGGACGAACGC)_2_ was used in all simulations and described with the parmbsc1 ^36^ force field. Octahedral boxes of at least 15 Å to the DNA were solvated with TIP3P water molecules,^37^ where SHAKE^38^ algorithm was applied to bonds involving hydrogens; sodium counterions together with a concentration of NaCl of 150 mM were added to the solvent. System equilibration was performed following the tutorial for DNA MD setup within the BioExcel Building Blocks software^39^ library. Within this framework, energetic minimization was followed with 15 ps of 1 fs time step at NVT ensemble with 5 kcal/mol heavy atomic restraints; afterward, three steps of energy minimization with 2 kcal/mol, 0.1 kcal/mol and without heavy atomic restraints were done. Thermalization steps 6, 7, and 8 consisted of NPT MD simulations with decreasing atomic restraints, 5 ps with 1 kcal/mol, 5 ps with 0.5 kcal/mol and 10 ps with 0.5 kcal/mol; step 9 was 10 ps of NPT without restraints and increasing time step to 2 fs and last step was a 1 ns-long repetition of step 9. Finally, production runs were carried out in triplicate with a duration of 5 μs, in which the time step was increased to 4f s, as hydrogen mass repartition^40^ was applied to the topologies with ParmEd.^41^ In the MD simulations, temperature was maintained at 310 K with Langevin dynamics^42^ with a gamma of 1.0 ps^–1^, and pressure isotropically at 1 atm with a Monte Carlo barostat.^43^ For the electrostatic interactions, a cutoff of 10 Å was applied using the particle-mesh Ewald method.^44^ All simulations were carried out in Amber software with GPU acceleration.^45^ Postprocessing was carried out mainly by means of CPPTRAJ^46^ scripts. Conversely, structural parameters of the DNA were obtained following the tutorial “Structural DNA helical parameters from MD trajectory tutorial using BioExcel Building Blocks (biobb)^39^” within the BioExcel Building Blocks software library, which uses a combination of Curves + ^47,48^ and Canal^47^ to automatically compute average of all structural parameters and their time series. Backbone RMSD Root Mean Square Deviation data are provided in the Supporting Information (Figures S7 and S10). Postprocessing scripts are available at Zenodo Repository.

### Nucleosome Core Particle Molecular Dynamics Simulations

The coordinates for nucleosome core particle systems were derived from previously reported simulations.^49^ Using the same methology,^49^ AMBER ff14SB^50^ was employed for the protein, parmbsc1 ^36^ for DNA and CUFIX^51^ ion parameter corrections were introduced.

The solvation box consists of an octahedral box with periodic boundary conditions of at least 1.2 nm away from the nucleosome atoms, filled with TIP3P water molecules,^37^ Na^+^ counterions to neutralize the system and 150 mM of Na^+^ and Cl^–^ ions. For system equilibration, minimization was followed by five consecutive NPT simulations reducing the heavy atom restraints, consisting in 100 ps with 0.5 fs time step with 500 kJ, and three 200 ps-long steps at 50 kJ, 5 kJ and 0.5 kJ, using a time step of 2 fs for the three thermalization steps; finally, 200 ps without constraints completed the thermalization. Afterward, three 5 μs-long production runs were considered for each simulated system. In these MD simulations, Langevin dynamics^41^ scheme was introduced to maintain the temperature at 300 K, and a Monte Carlo barostat^42^ for keeping the pressure at 1 atm. A cutoff of 8 Å^49^ was selected for electrostatic interactions, and the time step was kept at 2 fs. All simulations were carried out in Amber software with GPU acceleration.^43^ Postprocessing was carried out mainly by means of CPPTRAJ scripts, also available in our Zenodo Repository. The RMSD of the DNA backbone atoms is shown in Figure S15.

### Visualization and Rendering

Visualization and rendering of the structures were carried out with UCSF Chimera.^50^ and VMD software.^51^

## Results

### Structure and Origin of Chemical Reactivity in Aurkine16

To design a ligand able to generate interstrand cross-links,^19^ three key components were considered: a heterocyclic bidentate aromatic skeleton, an additional reactive site and a platinum­(II) dichloride metallic center (Figure 2A). The scaffold is synthesized via a double addition of 2-bromo-1-(pyridine-2-yl) ethan-1-one with methyl 2-aminoisonicotinate, followed by the addition of β-naphthyl group via palladium-catalyzed C–C coupling. The electrophilic carbon center, generated by the reduction of the ester group in the methyl 2-aminoisonicotinate side, is then added to the formed intermediate followed by the chloro-substitution of the alcohol group. A final reaction with platinum dichloride, complexed to equivalents of dimethyl sulfoxide (DMSO), takes place to deliver good yields of Aurkine16.^19^

**Figure 2:**
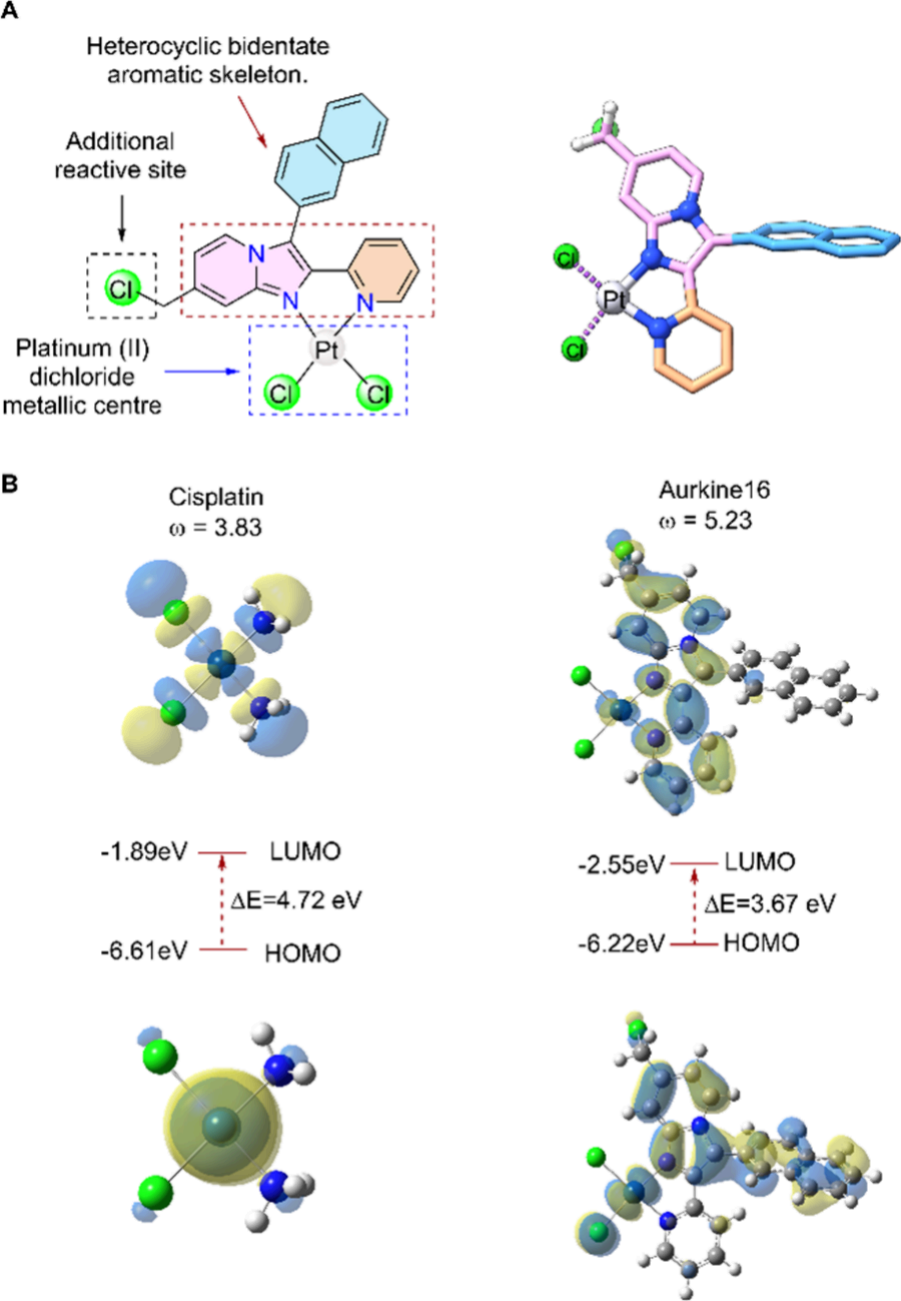
A, Structure of Aurkine16 and its key components. The heterocyclic bidentate aromatic skeleton is indicated by red dots, the platinum­(II) dichloride metal center is marked with black dots, and the additional reactive site is highlighted with gray dots. B, HOMO–LUMO energy gaps of Cis-Pt­(II) and Aurkine16. The electrophilicity is represented by the ω values in eV.

Optimization at DFT level of theory revealed interesting aspects of Aurkine16 compared to Cis-Pt­(II). Aurkine16 has a lower HOMO–LUMO energy gap, which is associated with softness and, consequently, higher chemical reactivity (Figure 2B and Figure S1). Indeed, the absolute softness calculated for the new platinum compound and Cis-Pt­(II) are 0.54 and 0.42, respectively (Table S2). Moreover, Aurkine16 demonstrates greater electrophilicity than cisplatin in both its neutral and monoaqua forms (Figure S1 and Table S2), a characteristic that may enhance its interactions with biological macromolecules such as DNA.^52^

The platinum metallic center can covalently bind to DNA, but before this attack happens, a first S_N_2 reaction takes place to activate the ligand through the entrance of a molecule of water, which acts as a nucleophile and displaces chloride. Configuration is retained as the product’s stereochemistry remains the same. The calculation of the Aurkine16 aquation reaction path (Figure 3) revealed preferential displacement of the chloride in the western position (structure **2** Figure 3). This is supported by a Gibbs activation energy that is 2.2 kcal/mol lower than that of the eastern position (structure **2a** Figure 3), corresponding to a reaction rate ratio of 3.86 in favor of the western side (See Section 2 in the Supporting Information).

**Figure 3:**
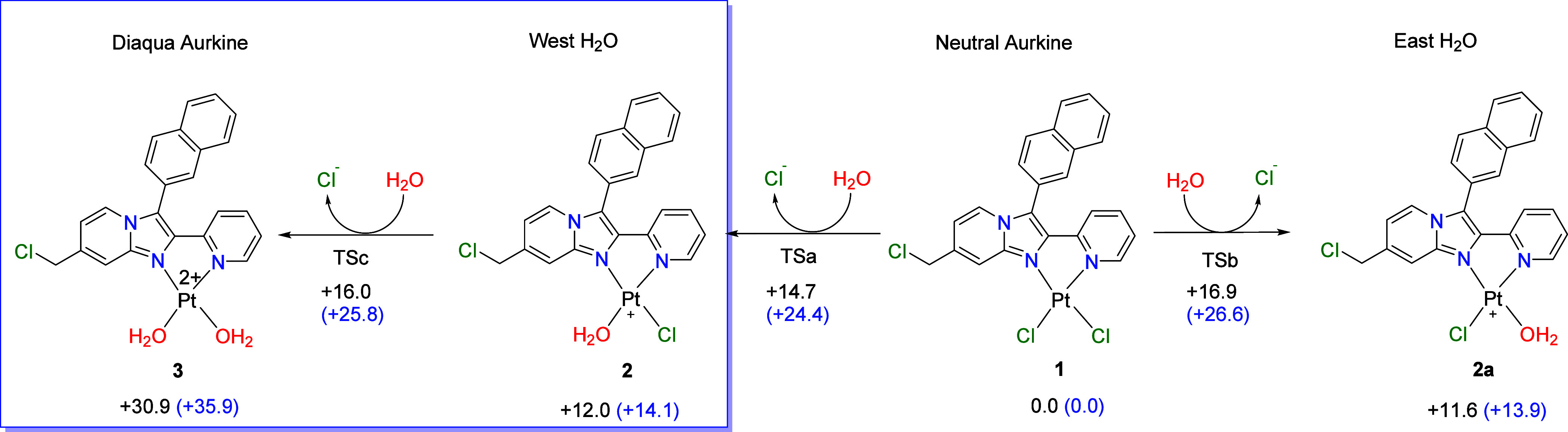
Activation and reaction energies (and Gibbs free energies in parentheses) of the reaction between Aurkine16 and water. All results were calculated at the B3LYP (PCM= water)/6–31+G**&LANL2DZ level of theory. Grimme’s D3 and Baker & Johnson (BJ) dispersion-corrected methods were considered. Values below structures or arrows correspond to relative or activation energies, respectively. Energy values are in kcal/mol.

From a geometrical standpoint, the entrance of a water molecule increases the Cl–Pt bond length in 0.43 Ȃ and forms an angle of 67.5° between the atoms O–Pt–Cl (Table S4) which results, as expected, in a pentacoordinate trigonal bipyramid^53^ platinum transition structure (TS) geometry (Figure S2). A second S_N_2 reaction, to turn the western monoaquated cation into the diaqua cation [Aurkine-2H_2_O]^+2^ (structure **3** in Figure 3), was carried out showing an activation barrier some extent higher than the monoaqua species.

Considering that the reaction of Aurkine16 with DNA occurs under physiological conditions, which can alter the equilibrium among species **1**, **2** and **3** (Figure 4), the rate constants^54^ associated with the formation of the mono- and diaqua cationic species were estimated using the Eyring equation. In this study, the activation Gibbs energy barriers (Table S6) were calculated by comparing the energies of stationary points directly connected through intrinsic reaction coordinate (IRC) calculations. With those results, the concentration of the Dichloride Aurkine16, western Monoaqua Aurkine16 and Diaqua Aurkine16 were then estimated by using numerical integration of the combined rate equations until the concentrations remained constant. Similarly to our previous work with Cis-Pt­(II),^55^ the initial concentrations of the Dichloride Aurkine16, [**1**]_0_, and water, [H_2_O]_0_, were 1 μM and 5.5x 10^7^μM, respectively. Also, two different concentrations of Cl^–^ were considered in the simulations to reproduce cytosolic ([Cl^–^] = 36 mM) and blood ([Cl^–^] = 102 mM) environments.^56^ The results of this analysis are gathered in Figure 5 and the script is also provided in the repository.

**Figure 4:**
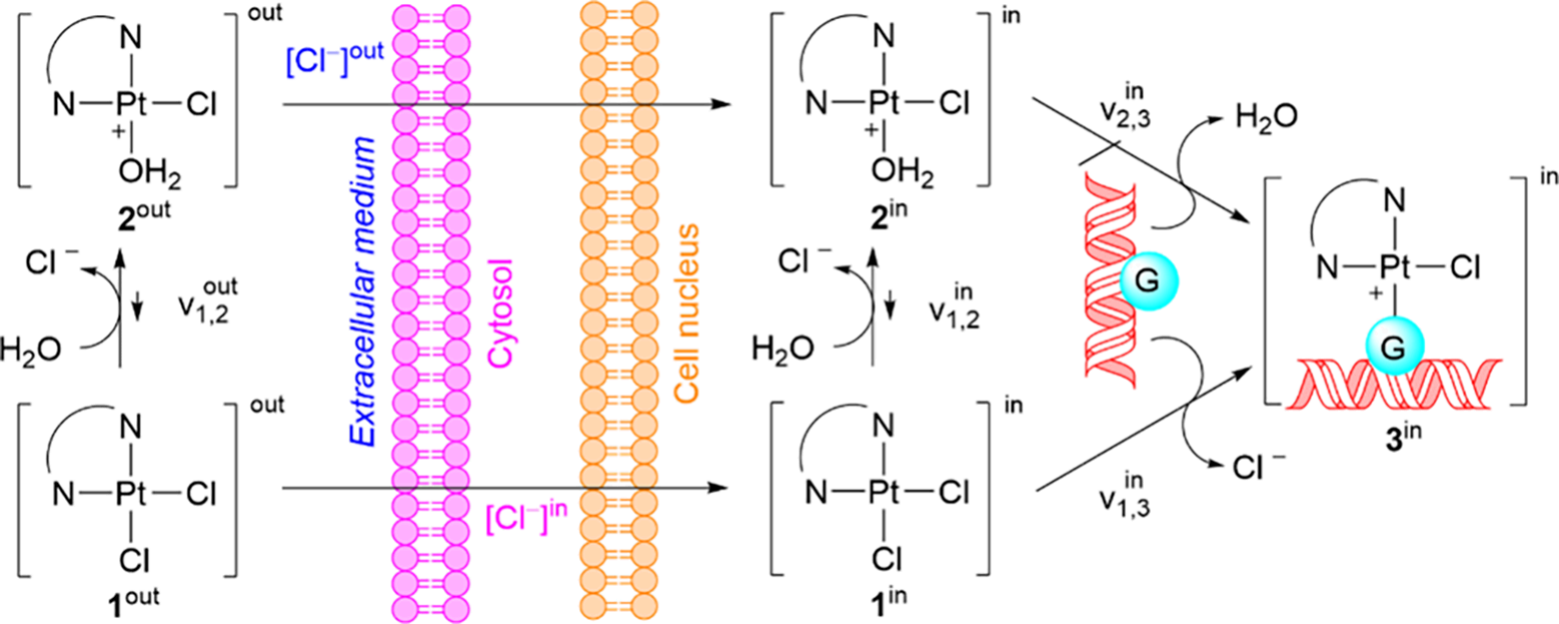
Schematic representation of potential pathways through the extracellular space, cytosol, and nucleus that lead to the transformation of neutral Aurkine16 (1) into the Aurkine16–DNA adduct (3) via monoaqua complex (2).

**Figure 5:**
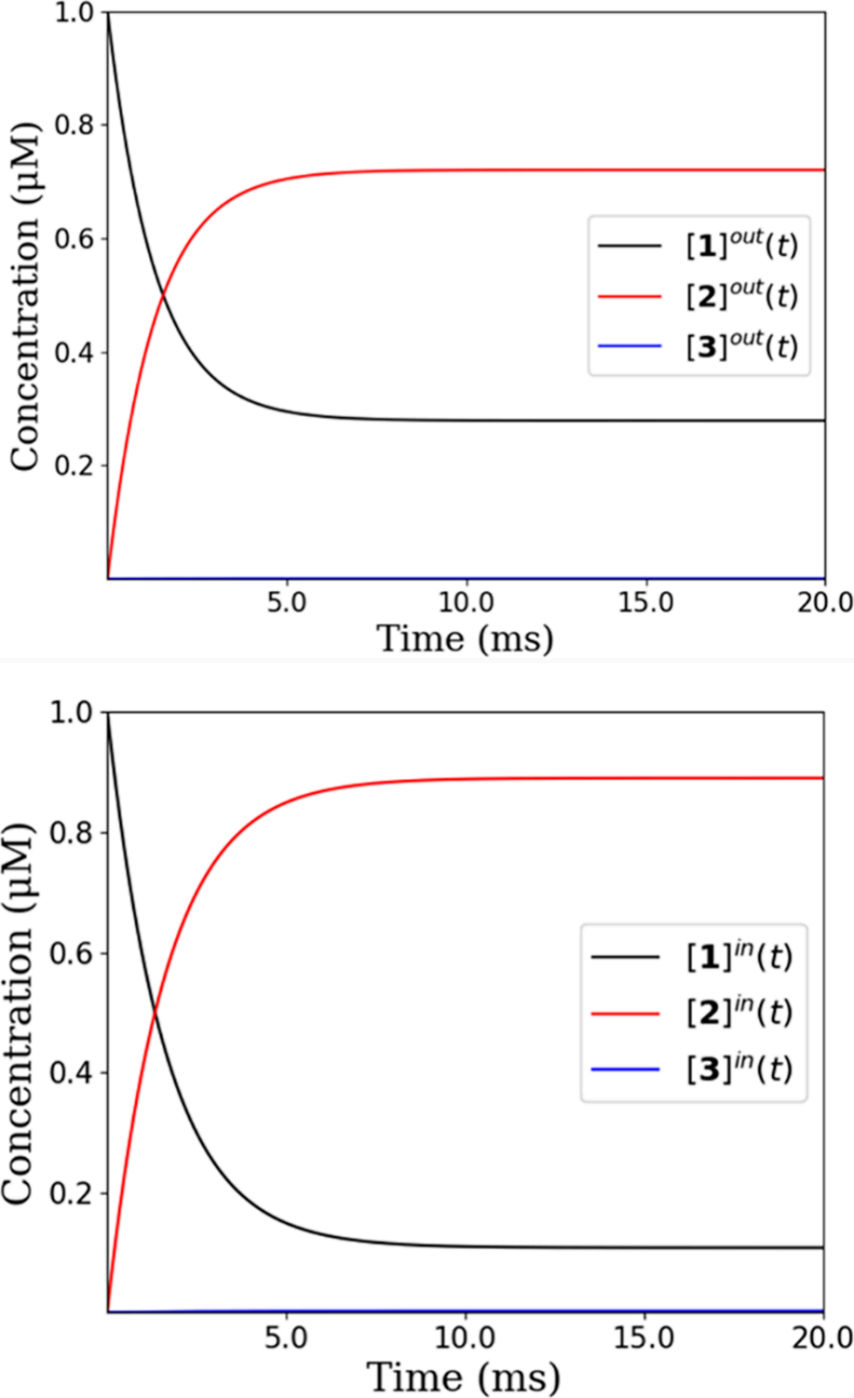
A, Simulated concentrations of Dichloride, Monoaqua and Diaqua Aurkine16 (structures 1,2 and 3 respectively) under blood conditions, this is, outside the cellular medium. B, Same for physiological conditions inside the cellular medium.

In the bloodstream, where the chloride concentration is higher, most of the Aurkine molecules are converted into the monocationic form, while approximately 35% of the platinum ligand remains in its original state ([**1**]^out^). However, inside the cytosol, where [Cl^–^] = 36 mM the conversion of dichloride ([**1**]^in^) to Monoaqua ([**2**]^in^) increases significantly, making the cytosol the most favorable environment for the monoaqua activation reaction. The formation of the Diaqua Aurkine16 ([**3**]^in^) was also observed, but its concentration is insignificant compared to that of the Monoaqua species.

These results suggest that under physiological conditions, Aurkine16 is primarily transformed after ca. 10 ms into a more reactive monoaqua cation, which can readily react with DNA nucleobases. Furthermore, there is a kinetic preference for this H_2_O molecule to be at the west position

### Microsolvation, Synchronicity, and Electrophilicity

A previous theoretical study on the reaction mechanism between Cis-Pt­(II) and purines^57–61^ demonstrated that during the activation reaction, water molecules act not only as competing nucleophiles but also as solvating agents, altering the characteristics of the chloride-leaving group.

This insight led us to investigate the aquation of Aurkine16, considering the presence of *n*-explicit water molecules in the reaction environment. This study focuses on the displacement of the western chloride, despite the energy difference being insufficient to fully neglect the eastern chloride’s displacement.

The results are presented in Table 1, and the transition structures associated with these reactions are depicted in Figure 6 and Figure S3. The addition of a single explicit water molecule has minimal effect on the activation energy (ΔEa) but increases the Gibbs free energy (ΔGa) by 1.6 kcal/mol. However, the introduction of a second water molecule reduces both ΔEa and ΔGa by 1.1 and 1.9 kcal/mol, respectively. Further additions of explicit water molecules continue to decrease ΔEa and ΔGa. The effect of a microsolvated environment on properties such as synchronicity (Sy)^57–61^ (see also Table S8 and Figure S4 in the Supporting Information for more details) and electrophilicity was also investigated. The results in Table 1 show that, generally, as synchronicity approaches one, ΔGa decreases. This also impacts electrophilicity, which tends to increase as the activation barriers decrease.

**Figure 6:**
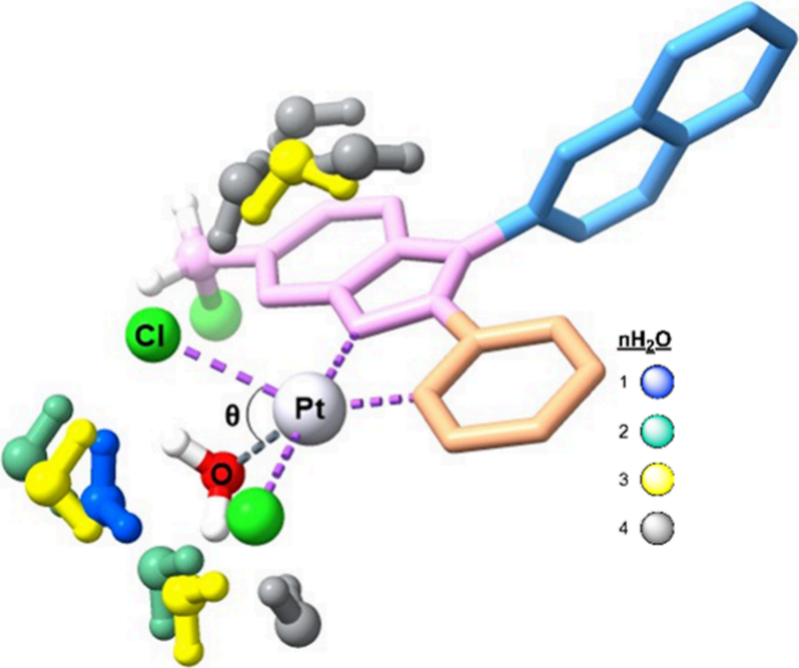
Superposition of the fully optimized transition structure of the aquation reaction considering the different numbers of explicit water molecules.

**Table 1:** Activation Energies, Gibbs Free Energies (kcal mol-1), Electrophilicity and Synchronicity of the Reaction between Aurkine16 and Water with Different Numbers of Additional Solvent Molecules (n)^a^

n	ΔEa	ΔGa	θ	ω^b^ (ev)	Sy^c^
0	+14.7	+24.4	66.7	5.23	0.84
1	+14.6	+26.1	66.7	5.27	0.85
2	+13.5	+24.2	66.2	5.28	0.84
3	+11.6	+22.8	65.7	5.39	0.90
4	+8.2	+21.8	65.7	5.42	0.98

a All results were calculated at the B3LYP-D3BJ­(PCM= water)/6-31+G***&LANL2DZ level of theory.

b Electrophilicity.

c Synchronicity.

### Reaction Mechanism

Our subsequent calculations focused on the S_N_2 reaction at the carbon site (Figure S5). In these QM simulations, we determined the activation barrier height for the reaction between Aurkine16 and G. This chemical transformation is characterized by a backside attack, which proceeds with the retention of configuration. The activation energies, Gibbs free energies, and geometric parameters of the transition structures are detailed in Figure S5. The covalent bond between Aurkine16 and Guanine involves the N7 position.

According to our calculations, 20.5 kcal/mol are necessary to G displace the chloride in the neutral Aurkine16 (Figure S5a). The evaluation of the S_N_2 reactions on carbon site of the Aurkine16 in its mono- and diaqua cationic forms indicates that the barrier heights for the western monoaqua Aurkine16 (Figure S5b) and the diaqua Aurkine16 (Figure S 5d) are 5.7 and 5.8 kcal/mol lower, respectively, than that of the eastern monoaqua cation (Figure S5c). With these results in mind, we must now exhaustively study the full reaction pathway to determine the least energetic route to the trisubstituted Aurkine-Guanine [Aurki-GGG]^3+^product. The reaction pathway leading to these products is illustrated in Figure 7. In the monocationic species **2**, water serves as a better leaving group than chloride, facilitating the entry of **G** to form the complex [Aurki-G]^+^ (**4**). Subsequently, the remaining chloride at the platinum site is substituted by a water molecule via saddle point **TS**_**8**_, thus giving rise to dicationic complex **6**. Another S_N_2 reaction follows to produce the disubstituted species [Aurki-GG]^+2^, followed by a carbon attack that yields the final trisubstituted product [Aurki-GGG]^3+^.

**Figure 7:**
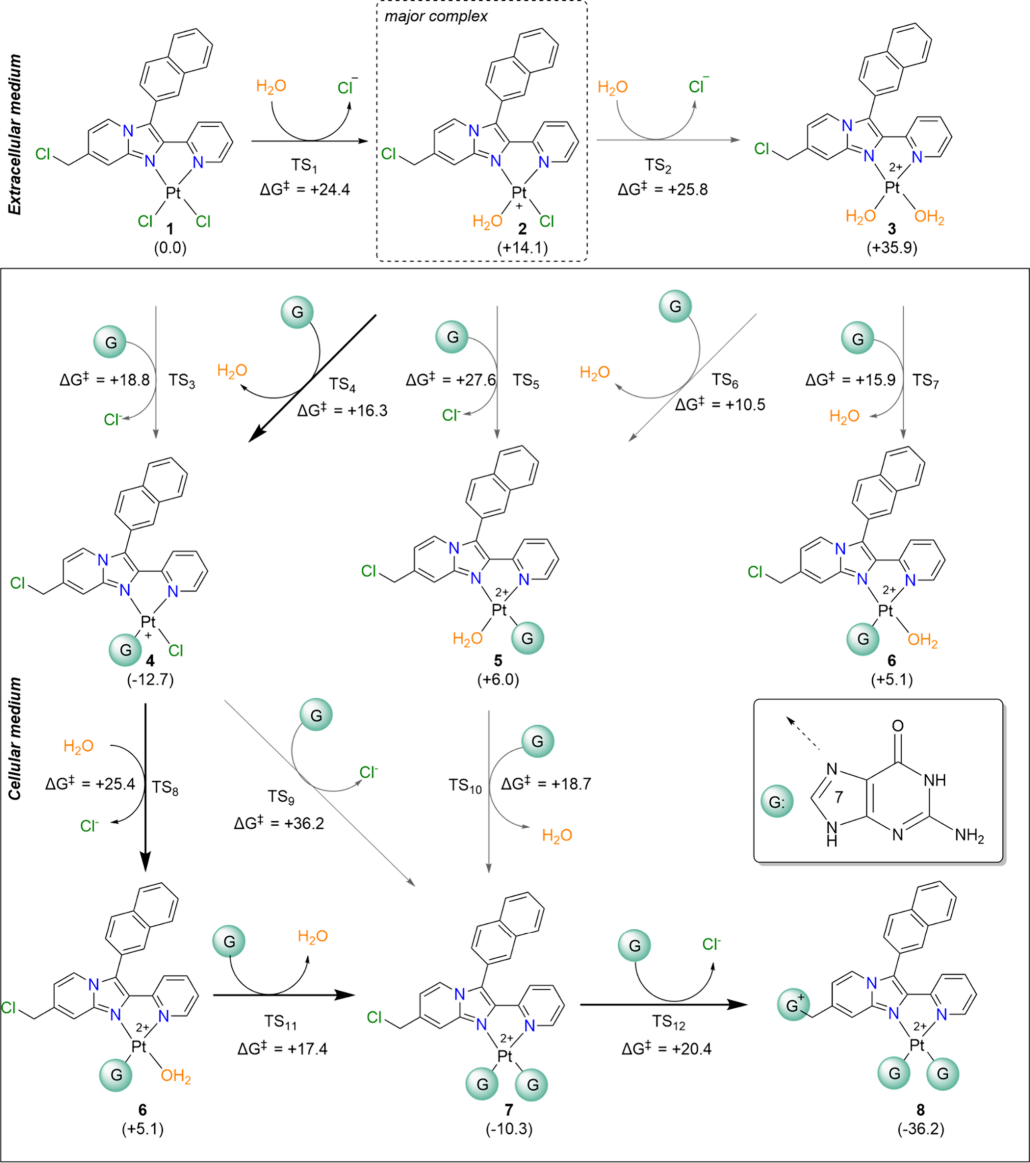
Reaction profile is associated with the reaction between Aurkine16’s and guanine molecules. Microsolvated environment was not considered. All results were calculated at the B3LYP-D3BJ­(PCM= water)/6–31+G**&LANL2DZ level of theory. Values below structures or arrows correspond to relative or activation energies, respectively. The relative Gibbs energies (in kcal/mol) were computed at 298.15 K. Red arrows indicate the energetically favorable pathway.

Although **TS**_**3**_ exhibits a lower activation energy than **TS**_**1**_—suggesting a preference for the formation of structure **4** over the monoaqua Aurkine16 species (structure **3**)—our previous calculations (vide supra) indicate that Aurkine16 predominantly exists in its monoaqua form under cytosolic conditions (Figure 5). Therefore, after activation, pathway **2 → 4 → 6 → 7 → 8** constitutes the dominant pathway for the formation of the trisubstituted Aurkine–Guanine cationic complex, as highlighted in Figure 7. To verify this preliminary conclusion, we performed numerical simulations under physiological conditions considering direct **1 → 4** transformation and **1 → 2 → 4** process. In these calculations, the rate constants derived from the Gibbs free energies in Figure 7 were used to compute the mole fraction of structure **4** over time for both possible pathways (Figure S6). To simulate the reaction conditions when Aurkine16 enters the nucleus, the initial concentrations were set to [Cl^–^]_0_ = 35 mM^56^ and [H_2_O]_0_ = 5.5 × 10^7^ μM. For guanine (G), considering that the DNA concentration in the nucleus is 10 mg/mL^62^ and that 41% of bases are GC pairs,^63^ the estimated starting concentration was [G]_0_ = 6.1 mM. The initial mole fractions for structures **1**, **2**, and **4** were [**1**]_0_^*in*^ = 0.88, [**2**]_0_^*in*^ = 0.11, and [**3**]_0_^*in*^ = 0, respectively, as computed in Figure 5. On the basis of the results gathered in Figure 4, in the DNA environment we obtain $\frac{{\left[2\right]}_{t}^{in}}{{\left[1\right]}_{t}^{in}}\approx 8.0$ and $\frac{v_{2\to 4}^{in}}{v_{1\to 4}^{in}}\approx 6.68$ . Under these conditions, our simulations indicate that the predominant pathway leading to the first attack of guanine is **1 → 2 → 4.**

In a previous study^19^ we investigated the effect of a microsolvated environment on this lower-energy pathway by introducing two water molecules near the reaction site. The results indicated that the microsolvated environment has a minimal impact on the formation of complex [Aurki-G]^+^ and [Aurki-GG]^2+^. However, the third nucleophilic substitution to form the complex [Aurki-GGG]^3+^ is significantly affected by the presence of water molecules, with the activation Gibbs free energy being 9.6 kcal/mol lower compared to a similar reaction step shown in Figure 7. Alternative routes for the Aurkine16-Guanine S_N_2 reaction were also considered (Figure S7). The pathway involving carbon attack by **G** after activation to form the monoaqua complex [Aurki-G]^+^ (structure **9** in Figure S7) exhibits a higher activation barrier compared to the S_N_2 attack where water serves as the leaving group via saddle point **TS**_**10**_ shown in Figure S7. Additionally, the sequence **2 → 9 → 11 → 13 → 8** (indicated by blue arrows in Figure S7) represents a competitive route, requiring only 4.2 kcal/mol of activation Gibbs free energy to form the [Aurki-GGG]^3+^ product (structure **8** in Figure S7).

After thoroughly exploring the Aurkine16-Guanine reaction pathway under various environmental conditions, we can conclude that the sequence **2 → 4 → 6 → 7 → 8** shown in Figure 7 represents the energetically most favorable route. All the alternative reaction pathways investigated in this work are found in Figure S7. However, their resultant activation barriers indicated a lower likelihood of occurrence, and therefore, those results have been omitted to streamline the discussion.

### Molecular Dynamics Calculations

In a previous study,^19^ we performed 5 μs of MD simulation to investigate the interaction of Aurkine16 with the 18-mer B-DNA 5′-G_1_C_2_A_3_C_4_G_5_A_6_A_7_C_8_G_9_G_10_A_11_C_12_G_13_A_14_A_15_C_16_

G_17_C_18_-3′ sequence. The most frequently observed binding modes were ligand intercalation, groove binding, and π-π stacking at terminal bases.

Among these binding modes, intercalation was identified as potentially disruptive to the DNA sequence. This process begins with the ligand reaching the DNA’s major groove, then increasing the distance between CpG base pair steps at the intercalation site, finally causing the DNA sequence to bend toward the minor groove. Additionally, as Aurkine16’s platinum site enters the intercalation pocket, the Pt–N7 distance decreases, exacerbating the DNA sequence distortion.

A deeper analysis of the simulation trajectory revealed that Aurkine16 follows these steps once in the intercalation site (Figure 8):

**1.** The ligand’s naphthyl group intercalates between the base steps without significantly disturbing the DNA sequence.
**2.** The ligand rotates, partially allowing the Pt site to enter the intercalation pocket.
**3.** The western side of the molecule, which contains the Cl attached to the carbon, fully enters the intercalation pocket, increasing DNA damage and remaining there until the end of the simulation.

**Figure 8:**
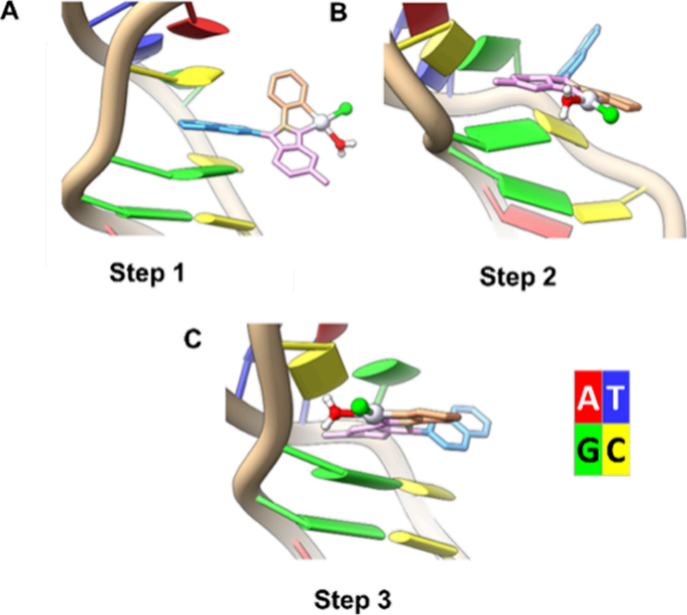
Representative steps in Aurkine16 intercalation: A, Step1: intercalation of the naphthyl group (in blue) between DNA base pairs. B, Step 2: ligand rotation enabling the Pt site to enter the intercalation pocket; C, Step 3: increased DNA damage resulting from the entry of the (ligand)­Pt­(OH_2_)Cl moiety (in green, red and pink) into the intercalation pocket.

### Aurkine16 Binding and Intercalating to an 18-mer B-DNA Sequence

Having a similar approach, the initial DNA-Aurkine16 box ensemble was thermalized and solvated to 5 μs MD simulations in triplicate, in order to expand the conformational space of the simulations. To do so, all-atom molecular dynamics (MD) simulations were performed on the same 18-mer sequence with Aurkine16 and Cis-Pt­(II) as ligands; initial conditions are depicted in Figure 9A,B, respectively. To identify the binding modes of both molecules, three 5 μs production runs were conducted.

**Figure 9:**
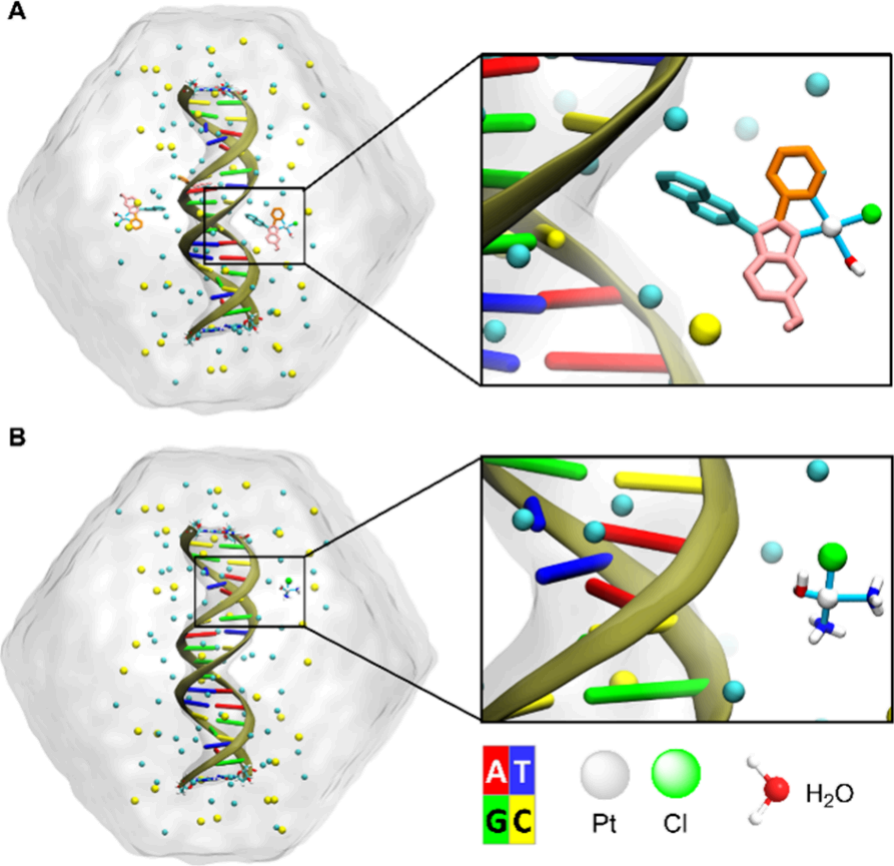
A, Initial conditions for the simulations of unbound simulations of Aurkine16, simulated sequence is 5′-G1C2A3C4G5A6A7C8G9G10A11C12G13A14A15C16G17C18–3′. B, same for CisPt.

Following established methods from the literature,^64^ three ligand molecules were introduced in the DNA-Aurkine16 simulations. This approach accounts for the tendency of Aurkine16’s aromatic rings to interact with DNA ends, which can result in nonrepresentative interactions due to the simplified nature of the simulated system, i.e., interactions that would not occur in a longer double-stranded DNA system.

Three-dimensional histograms showing the distribution of platinum atoms from Aurkine16 and Cis-Pt­(II) were generated through a density analysis, as shown in Figure 10A (left and right, respectively). For Cis-Pt­(II), most interactions occurred within the minor groove, with additional interactions involving the N7 of the GG base step, potentially indicative of typical Cis-Pt­(II) binding to these guanines. In the case of Aurkine16, two major density volumes were found at the DNA ends, due to expected π-stacking interactions between Aurkine16 and the DNA termini. Additionally, a significant binding hub was observed in the major groove around G9, and substantial density was observed in the minor groove.

**Figure 10:**
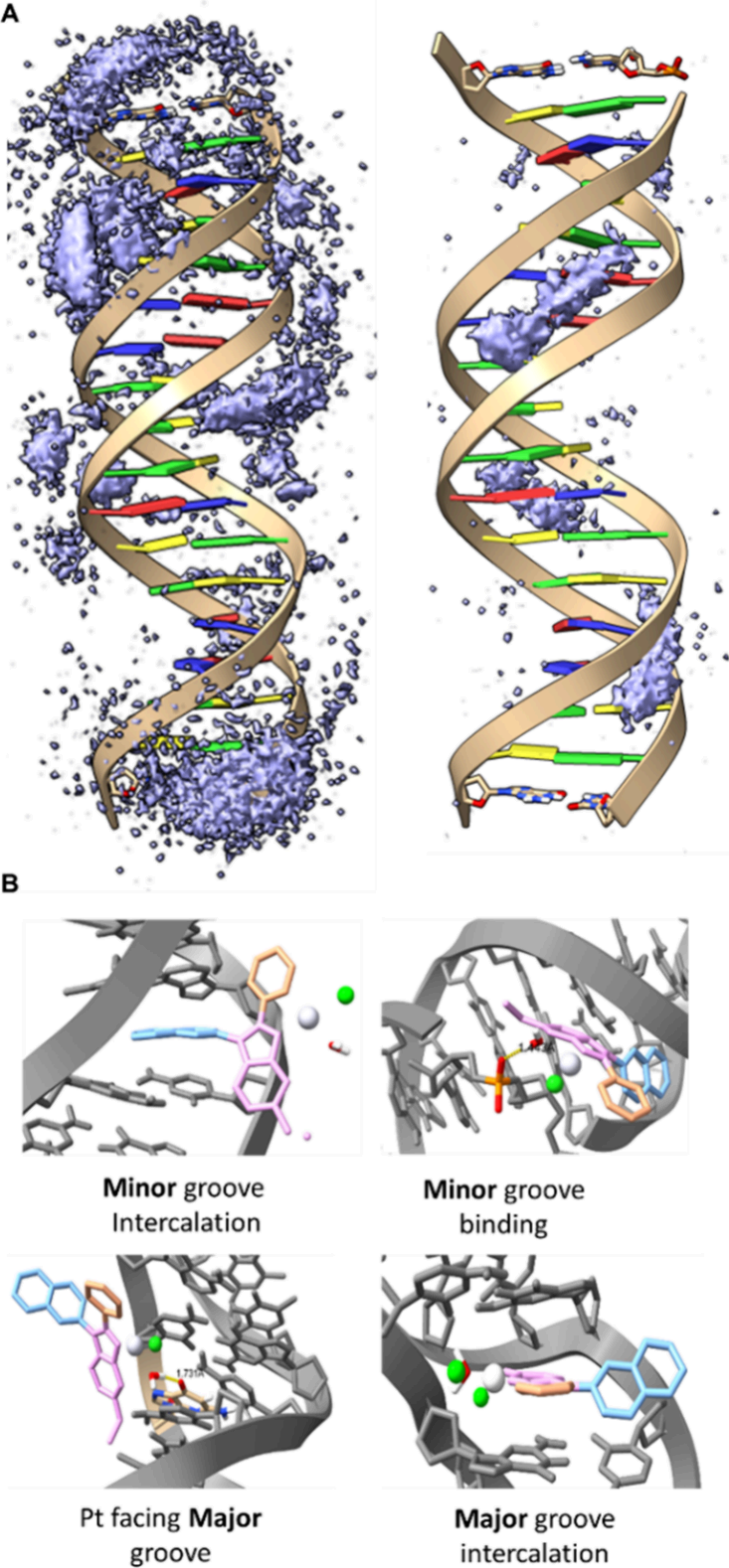
A, Grid density analysis of the Pt ion of the ligands in the unbound simulations of Aurkine16 (left) and CisPt (right). In both cases the same isodensity value was used. B, Different binding modes observed throughout Aurkine16 unbound simulations.

Visual inspection of the simulations revealed that Aurkine16, due to its greater structural complexity, exhibited multiple binding modes throughout the simulation (Figure 10B). Alongside Cis-Pt­(II)-like interactions in both the major and minor grooves—referred to as ″minor groove binding″ and ″Pt facing major groove″ in Figure 10B— Aurkine16 also demonstrated the ability to intercalate within both grooves, termed ″minor groove intercalation″ and ″major groove intercalation″ in Figure 10B. Notably, major groove intercalation occurred at 1 μs and persisted for the remainder of the simulation, as shown by the black line in Figure 11A-right, which represents the distance between the platinum atom of Aurkine16 and the N7 of G10. This distance is also illustrated as a black line in the system depiction in Figure 11A-right. Interestingly, the intercalation of Aurkine16 triggered a conformational change, evidenced by a significant increase in the RMSD of the DNA backbone atoms (Figure S8-Aurkine16’s first run), which rose from approximately 5Å to around 8Å; this abrupt change differentiated from the ones obtained in the other runs in the presence of the Aurkine16 or in the ones of the DNA without cofactors, in which they occur more gradually (Figure S8). To better understand this conformational change, we calculated the total bend angle using the software Curves+. Indeed, an increase in this angle was observed, coinciding with the intercalation of Aurkine16. Further details on the definition of the total bend can be found in the Supporting Information (Figure S9). The final snapshot of the trajectory, depicted in Figure 11A (right), visibly shows the bending of the DNA structure.

**Figure 11:**
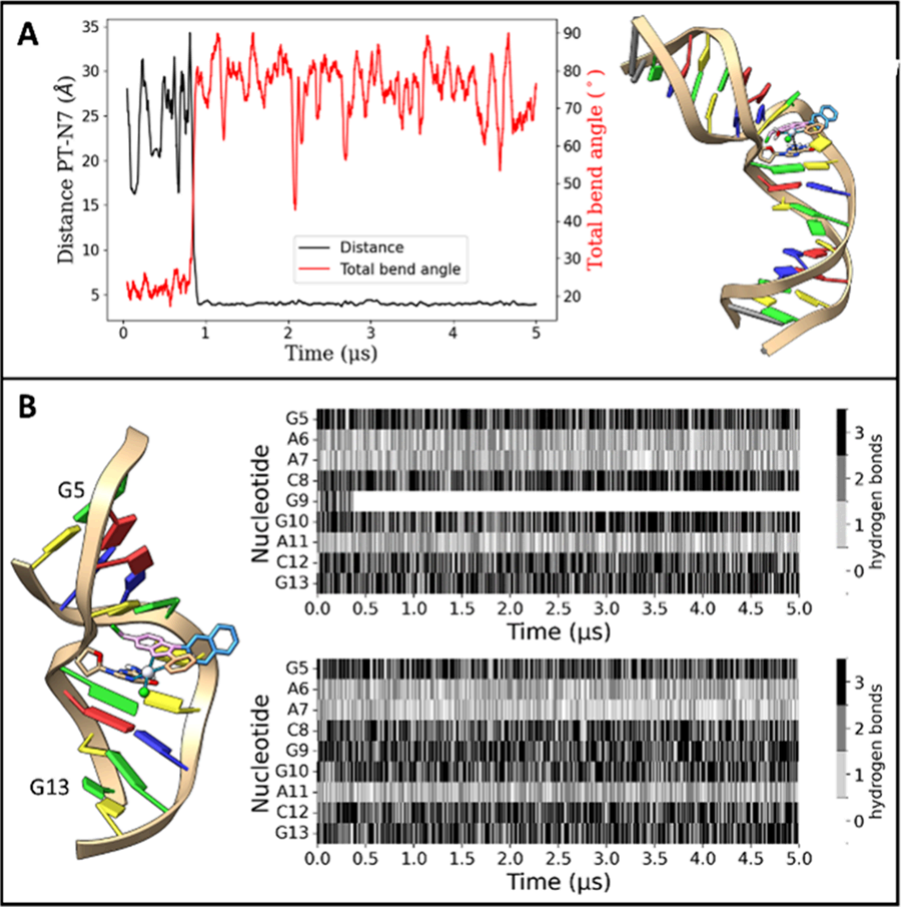
A, Analysis of the first run of the simulation of unbound Aurkine16, in which the intercalation takes place. Time series of the distance of the platinum ion of Aurkine16 and the reactive nitrogen (N7) of guanine G9 (black line),, and total bend angle of the DNA simulated fragment (red line, second axis). Depiction of the intercalation of the ligand and the structural impact on it with a black line showing the distance plotted (right). B, Initial conditions of the simulations of Aurkine16 intercalated with a covalent bond, reducing the depicted sequence to the bases G5-G13. (left), with the results of the WC-Hbond analysis of this reduced sequence for the bonded and intercalated simulation (right top) and the unbound simulation in which the intercalation takes place (right bottom).

Given the stability of this intercalation, we explored what would happen if the platinum atom subsequently attacked the N7 of G10, as seen with other platinum-based agents.^55^

To investigate this, we generated a new system that included a covalent bond between Aurkine16 and the N7 of G10 (Figure 11B-left) by taking the coordinates from the previous unbound simulation. We then analyzed the effect of this covalent bond by conducting a hydrogen bond analysis. Specifically, we computed the time series of Watson–Crick hydrogen bonds (WC-Hbonds) within the simulated sequence using an in-house CPPTRAJ script. For clarity, only the hydrogen bonds from G5 to G13 nitrogenous bases are shown in Figure 11B-left. The results of this analysis are shown in Figure 11B-top right: after a few hundred nanoseconds, all three WC-Hbonds are disrupted, leading to base pair eversion. This behavior was observed in all three simulation replicas (SI Figure S10). Interestingly, this result was unexpected, as intercalation from the free Aurkine16 simulations (system shown in Figure 9A) did not lead to base pair eversion (Figure 11B-bottom right). These findings suggest that when intercalation is not followed by covalent bonding, the system compensates by bending away from Aurkine16 to preserve base pair integrity.

### Aurkine16 Binding from a Cis-Pt­(II)-Like Mechanism

Considering that DNA binding is a crucial aspect of Aurkine16’s effect on DNA, we aimed to simulate the process by which Aurkine16 attacks DNA. As no crystal structures are available for this interaction, we proposed a Cis-Pt­(II)-like binding mechanism, which we studied through MD simulations. These simulations included various structural configurations for Aurkine16: one with a 1- intrastrand bond, one with a 1,2-intrastrand and another with a 3-’inter +1,2-intrastrand. For comparison, Cis-Pt­(II) with 1-intra and 1,2-intra bonds was also simulated, alongside a DNA reference without ligands. All simulated systems are depicted in Figure 12A.

**Figure 12:**
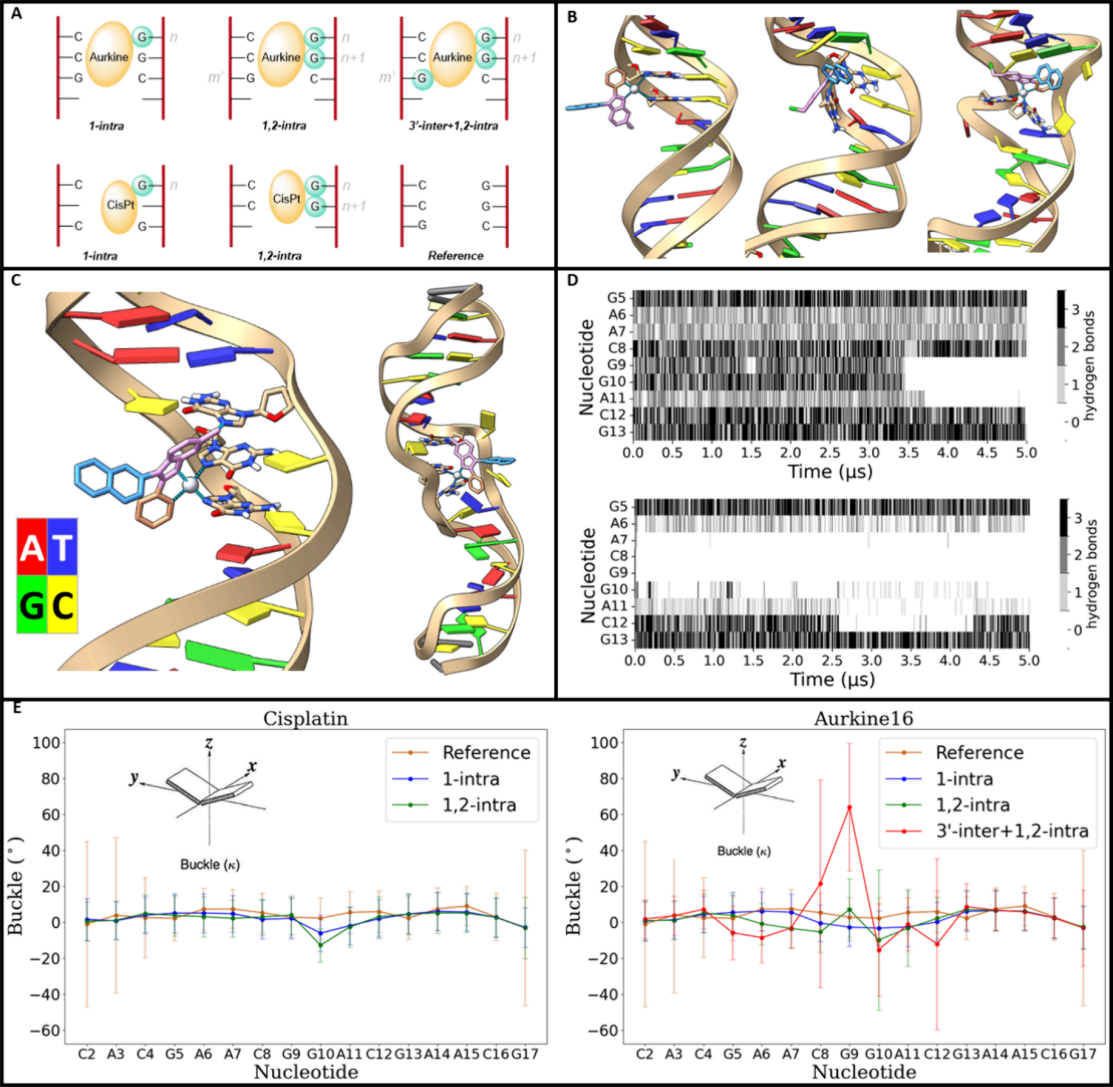
A, cartoon representation of the simulations carried out in this set of simulations, consisting in Aurkine16 covalently bound to one, two and three guanines, Cis-Pt­(II) bound to one and two guanines and the reference DNA without ligands. All of the simulations were run in triplicate with a time extension of 5 μs. B, intercalation process of Aurkine16 when covalently bound to two guanines. Starting confirmation of the simulation (left), π-π stacking interaction of the Aurkine16 with C8 (center) leading to the intercalation of the compound observed at the last frame of the simulation (right). C, starting conformation of the simulations of Aurkine16 covalently bound to three guanines in the 3′-inter+1,2-intra conformation (left) and final conformation of one of the three simulations (right). D, hydrogen bond analysis of the simulation depicted in B (top) and in C (bottom), in which intercalation of Aurkine16 takes place. E, average buck-le computed for each of the pair bases of the simulated systems, error bars account for the standard deviation of the computed values for all steps of the three replicas. Plots have been separated into Cis-Pt­(II) systems (left) and Aurkine16 systems (right) and depicted in both cases against the reference system. The calculation of the “buckle” structural parameter was carried out following the tutorial “Structural DNA helical parameters from MD trajectory tutorial using BioExcel Building Blocks (biobb)”.

The computational protocol followed the same methodology detailed in the previous section, which involved parameter generation for Aurkine16 and Cis-Pt­(II) with the different types of covalent bonds and three runs of 5 μs for each system. From a DNA-damage perspective, both the 1,2-intra bonded and the 3-’inter +1,2-intra bonded simulations exhibited intercalation, structural deformation, and base pair eversion.

In the case of the 1,2-intra bonded simulations, intercalation was observed in one of the three simulations. Key steps in this process are illustrated in Figure 12B. Specifically, the starting conditions for the 1,2-intra bonded simulations are shown in Figure 12B (left). Around 3.5 μs, the 2-pyridyl group of Aurkine16 (orange) began forming π-π stacking interactions with cytosine C8, inducing a distortion in the helical structure of the double-strand (Figure 12B-center). Eventually, Aurkine16 intercalated into the DNA, exacerbating the distortion and causing the eversion of bases G9, G10, and A11 (Figure 12B-right).

For the 3-’inter +1,2-intra bond simulations, whose initial conditions are depicted in Figure 12C (left), DNA damage was observed across all three simulations and was more pronounced, with several base pairs showing eversion and significant double helix distortion (Figure 12C-right). To track the timing of intercalation and to illustrate that the DNA damage was irreversible in the simulated time, we conducted a WC-Hbond analysis. The previously reported intercalation in the 1,2-intra bond simulation is shown in Figure 12D (top), alongside data from one of the 3-’inter +1,2-intra bond simulations (Figure 12D-bottom). Similar behavior was observed in the other 3-’inter +1,2-intra replicas (Figure S12), while no intercalation or pair eversion was detected in the other 1,2-intra replicas (Figure S13), nor in any of the 1-intra simulations (Figure S14) or those involving Cis-Pt­(II) (Figure S15).

In the following, we will focus on the effect of Cisp-Pt­(II) on the 18-mer DNA system; to do so, finer distortions in the base pairs were analyzed.^65^ Notably, a change in the helical parameter ″buckle″ was observed for Cis-Pt­(II), showing an increase in this structural parameter for the 1-intra bond simulations (Figure 12E-left: blue line) at G9 and G10 when compared to the reference (Figure 12E-left: orange line). This effect was even more pronounced in the 1,2-intra bond simulations (Figure 12E-left: green line), indicating that a greater number of covalent bonds exerted a stronger pull by the platinum ion, which is reflected in the buckle measurement.

For comparison with Aurkine16, the buckle was also computed for simulated systems containing this drug making 1 intra, 1,2-intra and 3′-inter+1,2-intra bonds (Figure 12E-right). The buckle from the 1-intra simulations (Figure 12E-right: blue line) was found to be similar to that of the Cis-Pt­(II) analogue. When more covalent bonds were included, the computed buckle increased as a direct consequence of base pair eversion; this was less pronounced in the 1,2-intra simulations (Figure 12E-right: green line), which exhibited a change in trend at G8, and was more significant in the 3-’inter +1,2-intra simulations (Figure 12E-right: red line).

### Aurkine16’s Behavior on the Nucleosome

To overcome the limitations associated with the simplicity of the 18-mer DNA system, we simulated Aurkine16 and Cis-Pt­(II) within a more complex environment: a nucleosome core particle. One of the key findings from experimental studies on Aurkine16 was its specificity toward cancer cells.^19^ In contrast, the toxicity of Cis-Pt­(II) has been attributed to off-target interactions, such as with histidine and methionine amino acids,^66^ as well as damage to healthy chromatin, as demonstrated by an X-ray crystal structure of 1,3-cis-{Pt­(NH_3_)_2_}^2+^-d­(GpTpG) intrastrand-bound platinated nucleosome.^67^ To investigate these concerns computationally, we conducted all-atom MD simulations of the entire nucleosome core particle (Figure 13A).

**Figure 13:**
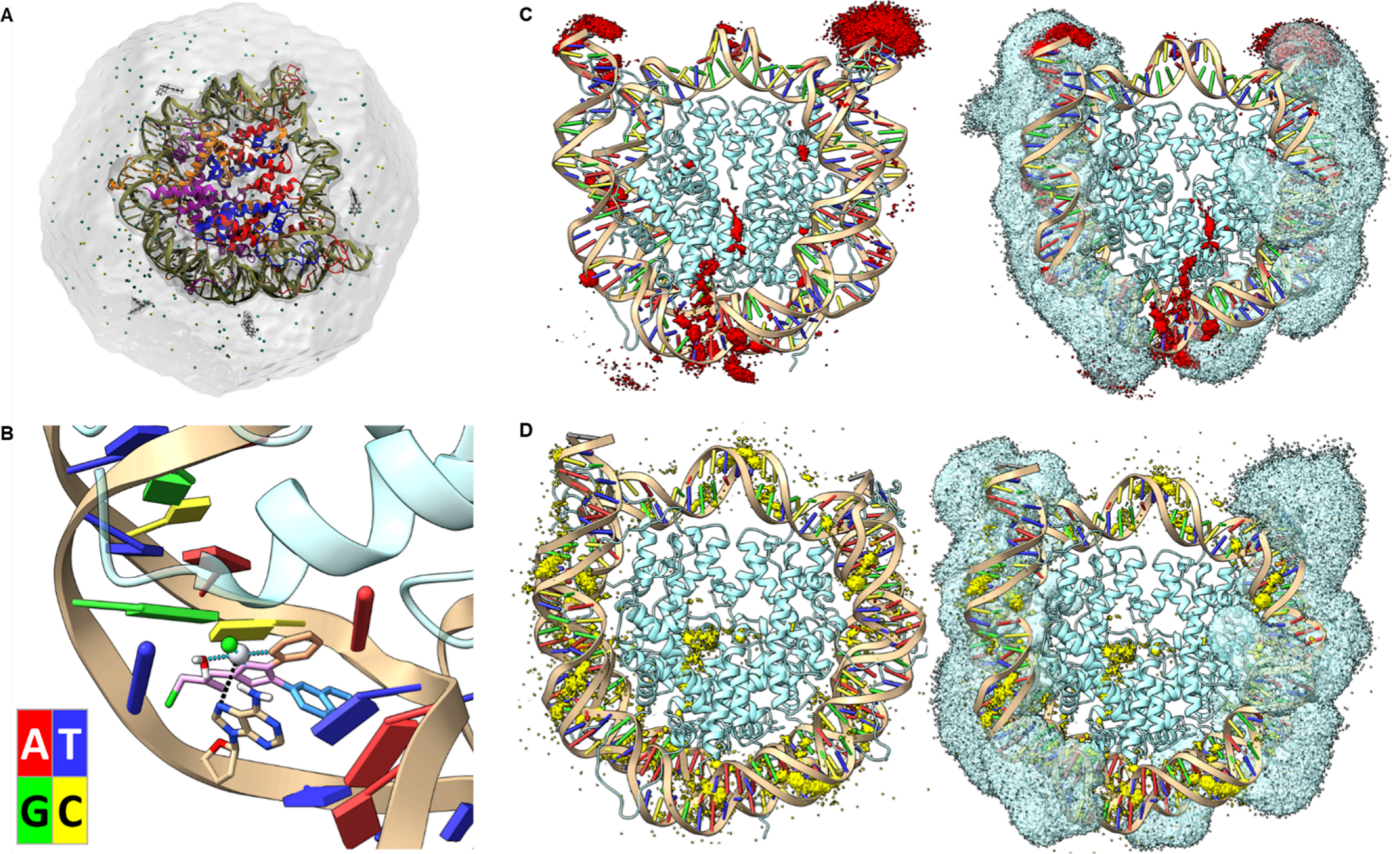
A, simulated system of the nucleosome, containing the two DNA strands in green and the different histone domains in red, blue, orange and purple, solvent is depicted as a gray surface, counterions and Aurkine16 are also visible. Similar systems were set up for Cis-Pt­(II) and reference without ligands. B, observed an intercalation event of Aurkine16 that leads to pair eversion. The distance of platinum and reactive nitrogen of adenine is highlighted in black. C, grid density analysis of platinum ion of Aurkine16 (red volume, left) and combined visualization of the densities of platinum and the histone tails (blue volume). D, same as C for Cis-Pt­(II).

The coordinates for our system were based on previously reported simulations of the nucleosome core particle^49^ (PDB ID: 1KX5 ^68^).

Visual inspection of the simulations revealed a significant intercalation of Aurkine16, as shown in Figure 13B. This interaction began with the 2-pyridyl group of Aurkine16 (orange), which subsequently rotated, allowing the platinum site to enter the intercalation pocket, similar to the intercalation observed in the unbound Aurkine16 simulations depicted in Figure 11A, this event happened around 700 ns and lasted for the rest of the simulated time. In all three simulations, additional intercalations of Aurkine16 occurred via the naphthyl group (blue) and the 2-pyridyl group (orange). However, these interactions proved unstable, as the ligand exited the intercalating pockets and returned to the solvent.

The interactions observed during the simulated time generated dense red volumes in the grid density analysis, shown in Figure 13C (left). Interestingly, these volumes did not overlap with the regions corresponding to the histone tails (Figure 13C-right: blue volume), suggesting that the histone tails exert steric pressure, which may help protect DNA from Aurkine16 interactions.

In contrast, the grid density analysis for Cis-Pt­(II) exhibited different behavior. The interactions were less localized, appearing consistently throughout the entire system (Figure 13D-left). Despite the presence of more stable interactions resulting in larger volume hubs, these interactions also did not coincide with the histone tails (Figure 13D-right: blue volume).

To quantify the potential attacks recorded during the simulations, we counted interactions between the platinum ions of the drugs and the N7 atoms of the DNA bases, defining an interaction as occurring when the distance was less than 4.5 Å. The results highlight the notable differences in the number of interactions for Aurkine16 (4 with adenines and 9 with guanines) compared to Cis-Pt­(II) (191 with adenines and 237 with guanines).

This indicates that Cis-Pt­(II) engages in approximately 80% and 70% of all possible interactions with guanines and adenines, respectively even though these intercalations are transient.

Finally, considering that Cis-Pt­(II) is capable of permeating the system and making more interactions than Aurkine16, we deemed it important to examine the number of off-target interactions that occurred during our simulations, particularly those involving histidine and methionine amino acids as the imidazole side group of histidine and the thiomethyl sulfur of metionines have been reported as the most favored platination sites among amino acids.^66^ A summary of these interactions is presented in Table S11. Cis-Pt­(II) exhibited a higher frequency of potentially toxic interactions with both types of residues, including some occurring within the histone domains. In contrast, throughout the simulated time Aurkine16 only showed a couple of interactions with histidine—one within the histone tails and another with a solvent-exposed residue.

## Discussion

Aurkines have been proposed as promising alternatives to conventional platinum-based chemotherapeutic agents for treating CCA.^19^ In this study, we present a comprehensive computational analysis of Aurkine16, investigating uptake upon physiological conditions, interaction with DNA guanine residues, dynamic behavior during DNA targeting, and performance within a more complex chromatin environment, such as the nucleosome core particle.

Given that CisPt is one of the most extensively studied platinum-based chemotherapeutic agents—both experimentally and computationally—it was used as a benchmark for comparative analysis. To date, even though some studies have analyzed the interactions between DNA and cisplatin,^69–71^ the dynamics of the attacking mechanism to the DNA has not been reported in the literature; therefore, we performed it for both Aurkine16 and CisPt.

Aurkine16 exhibits activation and cellular entry behaviors similar to other platinum-based drugs, relying on aquation for chemical activation. This process begins in the bloodstream, where the drug predominantly exists in the monoaqua [Aurkine-H_2_O]^+^ form (Figure 5). Activation is expected to be completed in the cytosol, where nearly the entire concentration is predicted to transition into the activated monoaqua form. Furthermore, our DFT calculations suggest that the aquation reaction is more likely to initiate at the western coordination site of Aurkine16 (Figure 3).

The pathways leading to the formation of the Aurkine-Guanine complex, which occurs once the molecule enters the cell, were thoroughly investigated at DFT level of theory. The study revealed the following sequence: initially, the [Aurki-H_2_O]^+^ cation is formed, and the western water molecule is displaced by guanine (G), resulting in the [Aurki-G]^+^ monocation. Subsequently, the eastern chloride is replaced by a water molecule, which is then displaced by a second guanine. Finally, the chloride attached to the carbon is displaced, leading to the formation of the [Aurki-GGG]^3+^ complex (Figure 7).

The literature widely agrees that CisPt induces single-strand DNA breaks, leading to cell cycle arrest, reduced cell proliferation, and eventual cell death.^11,12^ However, DNA repair mechanisms often restore the DNA, allowing cells to survive and evade cell death. In contrast, Aurkine exerts a more potent effect by inducing greater oxidative stress within the cell and mitochondria, activating caspases, and ultimately leading to enhanced cell death. Notably, Aurkines have demonstrated cytotoxic effects not only in treatment-naive CCA cells but also in CisPt-resistant cancer cells, including those from CCA, ovarian, and breast cancers. Mechanistically, this effect is attributed to Aurkine’s unique ability to induce double-strand DNA breaks at a higher frequency, in contrast to the single-strand lesions caused by CisPt.^19^

The theoretical study discussed here provides atomic-level insights to clarify these mechanisms. The computational analysis highlights two key differences between the drugs. First, Aurkine16 possesses a third electrophilic site, enabling it to simultaneously target three nucleic bases. Simulations of this interaction revealed that DNA integrity is entirely compromised, with both the double-helical structure and the surrounding Watson–Crick hydrogen bonds being disrupted (Figure 12). The second distinction is Aurkine’s ability to form π-π stacking interactions with nucleic bases via its naphthyl and 2-pyridyl groups. These interactions enable Aurkine to intercalate using its naphthyl group, either before initiating an attack or after the attack has occurred. In both scenarios, the intercalation induces base pair eversions and significant structural alterations to the DNA (Figure 11). These findings may explain the poor diffraction capability observed in the Aurkine-DNA complex. Furthermore, the shrinkage of the Pt–N7 distance from ca. 15 Å to ca. 5 Å in the latter stages of the preliminary interaction between DNA and Aurkine16 must induce, aside the structural distortion, a more kinetically advanced prenucleophilic interaction that must facilitate DNA damage in the SN2 process. Regarding the effect of CisPt on DNA observed in molecular dynamics simulations, we simulated 1-intra and 1,2-intrastrand cross-links, as *in vitro* experiments with Cis-Pt­(II) indicated these are the most probable interaction modes^18^ even though DNA lesions have also been associated with 1,3-intrastrand or 1–2’ interstrand bonds. However, simulating these more deleterious interactions requires DNA bending, for which we lacked appropriate starting configurations. From our simulations, the only structural effect observed was a slight distortion in the buckle angle, which aligns with structures reported in the literature.^22,72,73^ In particular, these changes are significant with respect to the ones observed for Cisplatin, which is a relevant reference.

Another promising advantage of Aurkines over CisPt is their selectivity for cancer cells.^19^ One plausible explanation for this difference comes from MD simulations of CisPt within the nucleosome core particle, which demonstrated CisPt’s ability to permeate the nucleosome core system, leading to significantly more off-target interactions. In contrast, during the simulated time Aurkine only interacts with the external residues of the histones, their tails, or DNA. Even though the different behavior that the two compounds exhibit in the simulations is clear, this could be amplificated from the inherent limitations of sampling in all-atom molecular dynamics simulations of complex systems. Nonetheless, we propose that the underlying mechanism for these differences in behavior may be related to the histone tails. These tails exert steric interactions that prevents larger compounds from permeating the system, making Aurkine16 more likely to interact with cancer cells, where chromatin is more accessible.

## Conclusion

The present study provides a detailed computational analysis of Aurkine16 as a promising alternative to platinum-based chemotherapeutic agents. By comparing Aurkine16 with the extensively studied CisPt, we identified key differences in their mechanisms of action. Both drugs share similarities in their activation process, relying on aquation for chemical activation. The reaction begins in the bloodstream, where most of the Aurkine16 is converted into its aqua monocationic form, with chloride displacement more likely to be initiated at the western site. The formation of the Aurkine-Guanine complex was thoroughly examined, revealing a series of displacements that lead to the formation of a stable [Aurki-GGG]^3+^ complex. The simulations showed in this work also revealed that Aurkine16’s ability to target multiple nucleic bases simultaneously and form π-π stacking interactions with DNA contributes to its potent cytotoxic effects, leading to significant structural alterations in the DNA.

Additionally, the study highlighted a critical advantage of Aurkine16 in terms of selectivity toward cancer cells. Unlike CisPt, which is capable of permeating the nucleosome core and leading to off-target interactions, Aurkine16 interacts specifically with external residues of the histones, their tails, or DNA, avoiding unintended interactions within the nucleosome core. This selectivity could be attributed to the steric interaction exerted by the histone tails, which restrict the penetration of larger compounds, making Aurkine16 more effective in targeting cancer cells where chromatin is more accessible.

Overall, the findings of this computational study provide significant insights into the distinct mechanisms of action of Aurkine16 and its potential as a more selective and effective chemotherapeutic agent compared to a traditional platinum-based drug.

## Supplementary Material



## Data Availability

The Cartesian coordinates, topologies, parameters and the python and bash scripts used in this paper are available at Zenodo Repository. These data include: (i) optimized geometries obtained after DFT calculations; (ii) postprocessing scripts to describe the interaction between Aurkine 16 and 18 mer DNA and nucleosome species; and (iii) molecular dynamics simulations (parameter folder, system generation and trajectories).

## Notes

Conceptual DFT-based properties; kinetics associated with aquation reactions; synchronicities; total energies, zero-point vibrational energy corrections, and thermal corrections of Gibbs energies; figures describing significant parameters of molecular dynamics simulations; off target contact analysis (PDF)
