# Supplementary material for: Computational Analysis of a Next-Generation Platinum-Based Chemotherapies that Induce DNA Double-Strand Breaks
Source: J Chem Inf Model. 2025 Nov 22;66(1):668–83. doi: 10.1021/acs.jcim.5c01654 (PMC12801317; doi:10.1021/acs.jcim.5c01654)
Supplement: Supplementary file 1 [file ci5c01654_si_001.pdf]

Supporting Information for

Computational Analysis of a Next-Generation Platinum-Based Chemotherapies that Induce DNA Double-Strand Breaks

Amanda R. Guimarães[a,b,*‡], Óscar R. Ballesteros[b,*‡], Iván Rivilla[a,b,c], Irene Olaizola[d], Mikel Odriozola-Gimeno[a], Abel de Cózar[a,c], David de Sancho[e], Xabier Lopez[e], Jesus M. Banales[c,d,f,g], Fernando P. Cossío[a,b,*]

[a]Department of Organic Chemistry I, Center of Innovation in Advanced Chemistry (ORFEO-CINQA), Faculty of Chemistry, University of the Basque Country (UPV/EHU), P° Manuel Lardizabal 3, 20018, Donostia/San Sebastián, Spain; [b]Donostia International Physics Center (DIPC), P° Manuel Lardizabal 4, 20018, Donostia/San Sebastian, Spain; [c]IKERBASQUE, Basque Foundation for Science, Mª Diaz de Haro 3, 48013, Bilbao, Spain; [d]Department of Liver and Gastrointestinal Diseases, Biogipuzkoa Health Research Institute - Donostia University Hospital -, University of the Basque Country (UPV/EHU), P° Dr. Begiristain, s/n, 20014, Donostia-San Sebastian, Spain; [e]Polimero eta Material Aurreratuak: Fisika, Kimika eta Teknologia & Donostia International Physics Center (DIPC), P° Manuel Lardizabal 3, 20018, Donostia/San Sebastian, Spain; [f]National Institute for the Study of Liver and Gastrointestinal Diseases (CIBERehd, "Instituto de Salud Carlos III") &Department of Biochemistry and Genetics, School of Sciences, University of Navarra, C/ Irunlarrea, 1. Pamplona 31008, Navarra, Spain.

*E-mail: amanda.ribeiro@ehu.es, oscar.rodriguez@dipc.org, fp.cossio@ehu.es

Table of Contents

1. Properties and Geometric parameters 5

Figure S1. HOMO-LUMO molecular orbitals (isovalue=0.02) of (A) the neutral cisplatin and Aurkine 16 end (B) the monoaqua cisplatin and Aurkine $16_{(west)}$ calculated at B3LYP (PCM= water)/6-31+G**& LANL2DZ level of theory. Grimme's D3 and Baker & Johnson (BJ) dispersion-corrected methods were considered. 6

Table S1- Total electronic energies (E, in Hartree), zero-point vibrational energies (ZPVE, in Hartree), Thermal corrections to Gibbs Free Energies (TCGFE, in Hartree), and number of imaginary frequencies (NIMAG) of all the structures in FigureS1. 6

Table S2. Properties calculated for the neutral cisplatin and Aurkine16 (A) and the monoaqua cisplatin and Aurkine 16 (B) Figure S1 (values are in eV). 6

Figure S2. Aurkine 16 aquation reaction pathway and transitions structures.....7

Table S3- Total electronic energies (E, in Hartree), zero-point vibrational energies (ZPVE, in Hartree), Thermal corrections to Gibbs Free Energies (TCGFE, in Hartree), and number of imaginary frequencies (NIMAG) of all the structures in Figure 3. 7

1.1 Relative Velocity Estimation from Activation Free Energies of the western and eastern aquation pathways. 7

Table S4. Geometrical properties of Aurkine 16 aquation reaction pathway depicted in Figure S2. Angles are in degree and the distance are in Å.................8

Table S 5 Total electronic energies (E, in Hartree), zero-point vibrational energies (ZPVE, in Hartree), Thermal corrections to Gibbs Free Energies (TCGFE, in Hartree), and number of imaginary frequencies (NIMAG) of the structures used to generate Figure 4..8

Table S6. Activation Free Energies (ΔG, in kcalmol^{-1}) and Kinetic Constants (ki→j, in M^{-1} s^{-1}), calculated at 298K, associated with the aquation reaction of aurkine16 in Figure 3. All results were calculated at the B3LYP (PCM= water)/6-31+G**&LANL2DZ level of theory. Grimme's D3 and Baker & Johnson (BJ) dispersion-corrected methods were considered. Gibbs energy barriers were determined by comparing the energies of stationary points directly connected through intrinsic reaction coordinate (IRC) calculations...................................8

Figure S3. Activation energies (and Gibbs free energies in parenthesis) of the reaction between the Aurki-Pt compound and water considering microsolvation. All results were calculated at the B3LYP (PCM= water)/6-31+G**&LANL2DZ level of theory. Grimme's D3 and Baker & Johnson (BJ) dispersion-corrected methods were considered. Values are in kcalmol-1. Transition structures are on the top of the arrows...9

Table S7 - Total electronic energies (E, in Hartree), zero-point vibrational energies (ZPVE, in Hartree), Thermal corrections to Gibbs Free Energies (TCGFE, in Hartree), and number of imaginary frequencies (NIMAG) of all the structures in Figure S3. 9

Table S8. Properties calculated at DFT level of theory for Aurkine16 within different microsolvation environment. 10

Figure S4. Variation of the electrophilicity with the quantity of explicit water molecules. 11

2. Reaction pathways analysis 12

Figure S5. Reaction profile associated with the displacement of the Aurkine16's chloride, attached to the carbon, by a guanine molecule. Microsolvated environment was not considered. All results were calculated at the B3LYP (PCM= water)/6-31+G**&LANL2DZ level of theory. Grimme's D3 and Baker & Johnson (BJ) dispersion-corrected methods were considered. Values below structures or arrows correspond to relative or activation Gibbs energies, respectively (in kcal/mol) computed at 298.15 K..12

Table S9 – Total electronic energies (E, in Hartree), zero-point vibrational energies (ZPVE, in Hartree), Thermal corrections to Gibbs Free Energies (TCGFE, in Hartree), and number of imaginary frequencies (NIMAG) of all the structures in Figure S5. 13

Figure S6. Map containing all the routes calculated in this work to reach the [Aurki-GGG]$^{3+}$ product. 14

Table S10 - Total electronic energies (E, in Hartree), zero-point vibrational energies (ZPVE, in Hartree), Thermal corrections to Gibbs Free Energies (TCGFE, in Hartree), and number of imaginary frequencies (NIMAG) of all the structures in Figure S6. 15

3. Molecular Dynamics Simulations 16

Figure S7. Root Mean Square Deviation of the DNA oligomer throughout the simulation time for the three runs performed (black, green, and blue lines, respectively), corresponding to the simulations described in the section *Aurkine16 Binding and Intercalation to an 18-mer B-DNA Sequence*. Specifically, these simulations include the DNA oligomer without cofactors (reference), the DNA oligomer in the presence of Cis-Pt(II) and Aurkine16 (Aurkine), and the specific case where Aurkine16 is covalently bonded and intercalated (1-intra). 16

Figure S8. Illustration of the total bend angle calculation, defined as the angle formed by the vectors u and v, which are normal to planes π1 and π2. For this calculation, the edge residues at the 5' and 3' ends were excluded. Further details on the definition of the planes can be found in reference [9]. 17

Figure S9. Hydrogen bond analysis for Aurkine16 intercalated with the 1-intra covalent bond across the three simulated replicas. The number of Watson-Crick hydrogen bonds was calculated throughout the simulation time. The CPPTRAJ code used for this analysis is provided below. 18

Figure S10. Root Mean Square Deviation of the DNA oligomer over the simulation time for the three performed runs (represented by black, green, and blue lines, respectively). This figure corresponds to the simulations described in the section *Aurkine16 binding via a CisPt-like mechanism*, which include Cisplatin with one and two covalent bonds (1-intra and 1,2-intra, respectively) and Aurkine16 with one, two, and three covalent bonds (1-intra, 1,2-intra, and 3'-inter+1,2-intra, respectively). A depiction of the simulated systems can be found in Figure 11A of the main text. 18

Figure S11. Hydrogen bond analysis for Aurkine16 3'-inter+1,2-intra bound to DNA across the three simulated replicas. The number of Watson-Crick hydrogen bonds was calculated throughout the simulation time. The CPPTRAJ code used for this analysis is provided below. 19

Figure S12. Hydrogen bond analysis for Aurkine16 1,2-intra bound to DNA across the three simulated replicas. The number of Watson-Crick hydrogen bonds was calculated throughout the simulation time. The CPPTRAJ code used for this analysis is provided below. 20

Figure S13. Hydrogen bond analysis for Aurkine16 1-intra bound to DNA across the three simulated replicas. The number of Watson-Crick hydrogen bonds was

calculated throughout the simulation time. The CPPTRAJ code used for this analysis is provided below.........21

Figure S14. Hydrogen bond analysis for Cisplatin 1-intra bound to DNA (left) and Cisplatin 1,2-intra bound to DNA across the three simulated replicas. The number of Watson-Crick hydrogen bonds was calculated throughout the simulation time. The CPPTRAJ code used for this analysis is provided below.........22

Figure S15. Root Mean Square Deviation of the DNA in the nucleosome core particle throughout the simulation time for the three runs performed (black, green, and blue lines, respectively). This relates to the simulations described in the section *"Aurkine16's Behaviour on the Nucleosome"*, which involve the nucleosome core particle (reference: PDB 1KX5) in the presence of four Aurkine16 molecules (Aurkine16) and four Cisplatin molecules (CisPt). Aurkine16 is depicted in Figure 12 of the main text.........22

Table S11 - Off-target contact analysis of Pt ions belonging to Cis-Pt(II) and Aurkine16. Each observed contact is described with the residue index in our system and the location based on the pdb-based convention of (PDB ID: 1KX566). For each contact, the percentage of frames in which at least one Pt ion is in contact (less than 4.5 Å) is provided together for both Cisplatin and Aurkine16 simulations, together with a comment on the specific location of the amino acid. The contact fractions have been computed with an in-house provided in the supporting information. 23

1. Properties and Geometric parameters

To calculate the electronic properties Electronegativity (χ)[1,2], Absolute Hardness[1,2] (η), Chemical Potential (π)[2], Absolute Softness (σ)[1,2], and Electrophilicity (ω)[3], we use molecular orbital energy values derived from the DFT calculations of the HOMO-LUMO energy gaps displayed in Figure S1

a. Electronegativity (χ)

$$\chi = \frac{(E_{HOMO}+E_{LUMO})}{2} \qquad 1$$

Here, E_{HOMO} and E_{LUMO} are the energies of the HOMO and LUMO, respectively.

b. Absolute Hardness (η)

$$\eta = \frac{(E_{LUMO}-E_{HOMO})}{2} \qquad 2$$

A larger η value indicates greater stability and resistance to deformation in electron density.

c. Chemical Potential (π)

The chemical potential describes the escaping tendency of electrons from a system. It is simply the negative of electronegativity:

$$\pi = -\chi \qquad 3$$

d. Absolute Softness (σ)

Absolute softness is the inverse of absolute hardness:

$$\sigma = \frac{1}{\eta} \qquad 4$$

A higher σ value indicates greater polarizability and reactivity.

e. Electrophilicity Index (ω)

The electrophilicity index quantifies the propensity of a molecule to accept electrons. It is defined as:

$$\omega = -(\pi^2/2\eta) \qquad 5$$

A higher ω value signifies a stronger electrophile, meaning it is more likely to attract electrons from nucleophiles.

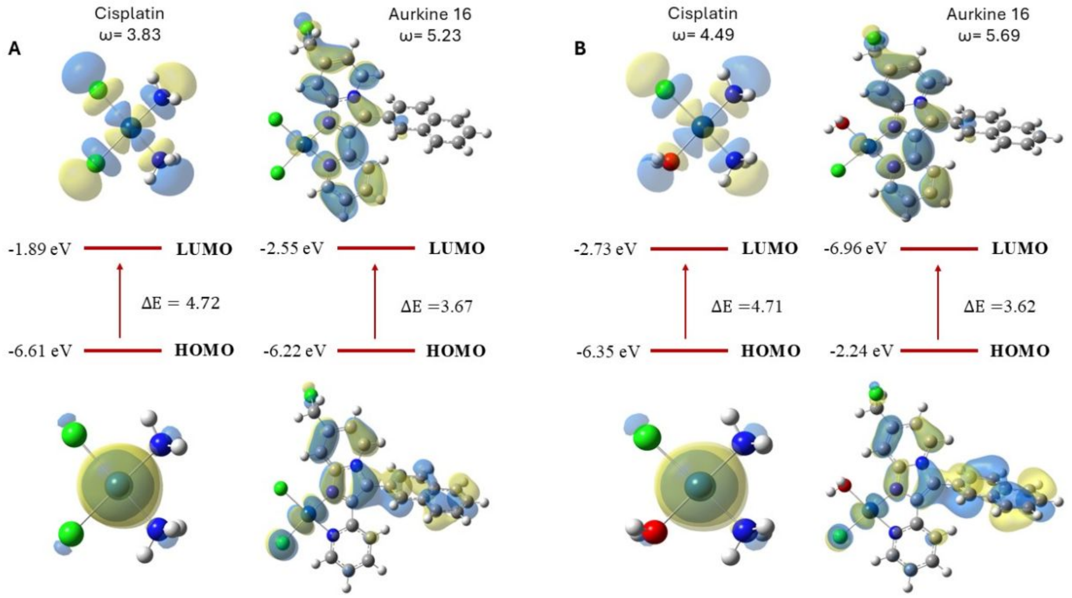

Figure S1. HOMO-LUMO molecular orbitals (isovalue=0.02) of (A) the neutral cisplatin and Aurkine 16 end (B) the monoaqua cisplatin and Aurkine $16_{(west)}$ calculated at B3LYP (PCM= water)/6-31+G**& LANL2DZ level of theory. Grimme's D3 and Baker & Johnson (BJ) dispersion-corrected methods were considered.

Table S1- Total electronic energies (E, in Hartree), zero-point vibrational energies (ZPVE, in Hartree), Thermal corrections to Gibbs Free Energies (TCGFE, in Hartree), and number of imaginary frequencies (NIMAG) of all the structures in FigureS1.

FigureS1	Structure	Energy	ZPCE	TCGFE	NIMAG(ν)
**A**	Cis-Pt(II)	-1152.872398	0.082530	0.046837	0
	Aurkine neutal	-2550.446145	0.340090	0.280715	0
**B**	monoaqua Cis-Pt(II)	-768.915123	0.107722	0.071677	0
	monoaqua Aurkine	-2550.079437	0.340090	0.280715	0

Table S2. Properties calculated for the neutral cisplatin and Aurkine16 (A) and the monoaqua cisplatin and Aurkine 16 (B) Figure S1 (values are in eV).

	**Neutral**		**Monoaqua**	
**Property**	Cis-Pt(II)	Aurkine16	Cis-Pt(II)	Aurkine 16 $_{(west)}$
HOMO-LUMO energy gap	4.72	3.67	4.71	3.62
Electronegativity (χ)	4.25	4.39	4.60	4.54
Absolute Hardiness (η)	2.36	1.84	2.36	1.81
Chemical Potential (π)	-4.25	-4.39	-4.60	-4.54
Absolute softness (σ)	0.42	0.54	0.42	0.55
Electrophilicity (ω)	3.83	5.23	4.49	5.69

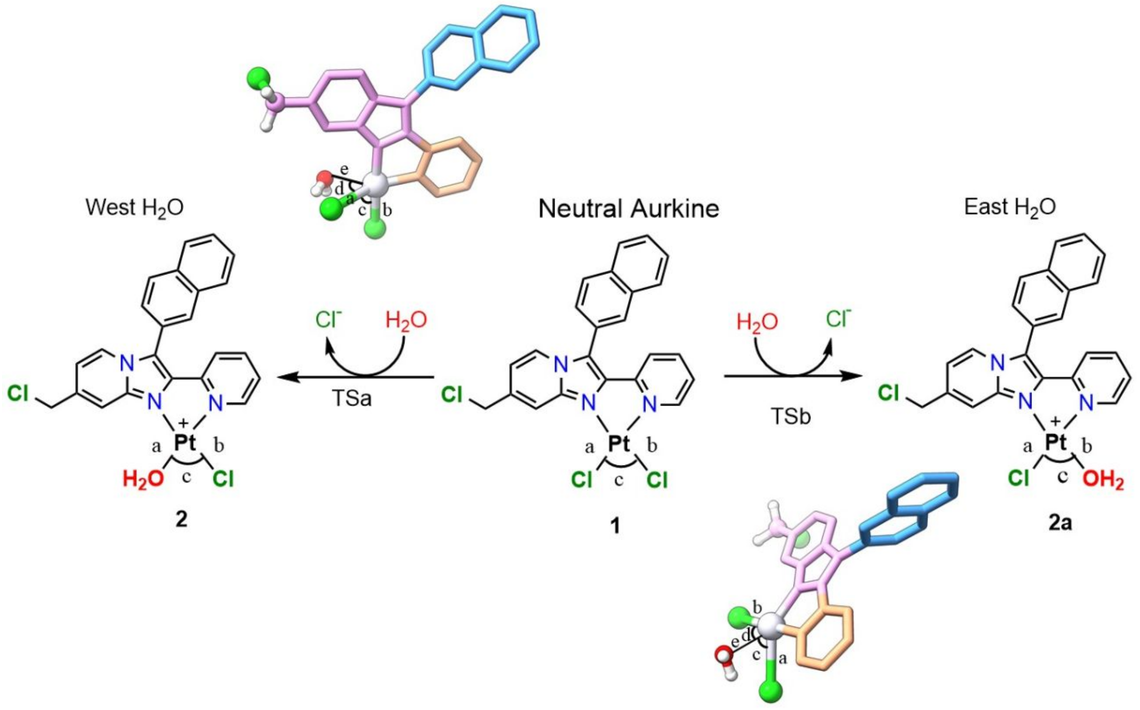

Figure S2. Aurkine 16 aquation reaction pathway and transitions structures.

Table S3- Total electronic energies (E, in Hartree), zero-point vibrational energies (ZPVE, in Hartree), Thermal corrections to Gibbs Free Energies (TCGFE, in Hartree), and number of imaginary frequencies (NIMAG) of all the structures in Figure 3.

Structure	Energy	ZPCE	TCGFE	NIMAG(ν)
1	-2550.446145	0.340090	0.280715	0
2	-2166.490534	0.365175	0.305712	0
2a	-2166.491382	0.365320	0.306217	0
3	-1782.524223	0.390662	0.332188	0
TS_1	-2626.866931	0.364040	0.302027	1(-159.9805)
TS_{1a}	-2626.863429	0.364096	0.302029	1(-164.0242)
TS_2	-2242.909316	0.389356	0.327262	1(-162.9875)

1.1 Relative Velocity Estimation from Activation Free Energies of the western and eastern aquation pathways.

The velocity ratio between the western and eastern activation pathways was determined using the following expression derived from transition state theory:

$$\frac{v_{west}}{v_{east}} = \exp - \left(\frac{\Delta G^{\ddagger}_{west} - \Delta G^{\ddagger}_{east}}{RT} \right)$$

where R is equal 0.001987 kcal mol^{-1} K^{-1} and T= 298 K. Here, $\Delta G^{\ddagger}_{west}$ and $\Delta G^{\ddagger}_{east}$ denote the activation Gibbs free energies for the western and eastern monoaquation reactions, respectively.

$$\frac{k_{west}}{k_{east}} = \exp - \left(\frac{25.8 - 26.6}{0.5927}\right) = 3.86$$

Table S4. Geometrical properties of Aurkine 16 aquation reaction pathway depicted in Figure S2. Angles are in degree and the distance are in Å.

Property	Reactant	TSa	TSb	Product west	Product east
a (Pt-Cl)	2.39	2.82	2.36	-	2.39
b (Pt-Cl)	2.39	2.39	2.86	2.38	-
a (Pt-H_2O)	-	-	-	2.14	-
b (Pt-H_2O)	-	-	-	-	2.14
e (Pt-H_2O)	-	2.49	2.49	-	-
c(∡Cl-Pt-Cl)	87.8	91.0	90.4	-	-
d(∡H_2O-Pt-Cl)	-	67.5	66.8	85.0	85.5

Table S5 Total electronic energies (E, in Hartree), zero-point vibrational energies (ZPVE, in Hartree), Thermal corrections to Gibbs Free Energies (TCGFE, in Hartree), and number of imaginary frequencies (NIMAG) of the structures used to generate Figure 4.

Process	Structure [a]	Energy	ZPCE	TCGFE	NIMAG(v)
1→2<br>1←2	RC_1	-2626.890124	0.363545	0.299537	0
	TS_1	-2626.866931	0.364040	0.302027	1(-159.9805)
	PC_2	-2626.887148	0.365099	0.301548	0
2→3<br>2←3	RC_2	-2242.939475	0.389563	0.326441	0
	TS_2	-2242.909316	0.389356	0.327262	1(-162.9875)
	PC_2	-2242.929389	0.390735	0.329005	0

[a]RC = Reactant complex, PC=Product Complex

Table S6. Activation Free Energies (ΔG, in kcalmol^{-1}) and Kinetic Constants ($k_{i\to j}$, in M^{-1} s^{-1}), calculated at 298K, associated with the aquation reaction of aurkine16 in Figure 3. All results were calculated at the B3LYP (PCM= water)/6-31+G**&LANL2DZ level of theory. Grimme's D3 and Baker & Johnson (BJ) dispersion-corrected methods were considered. Gibbs energy barriers were determined by comparing the energies of stationary points directly connected through intrinsic reaction coordinate (IRC) calculations.

Process	ΔG‡	k_{ij}
1→2	+16.1	1.0E+01
1←2	+13.0	1.9E+03
2→3	+19.0	7.7E-02
2←3	+11.0	5.5E+04

The kinetic constant values were obtained via

$$k = \frac{K_B T}{h} e^{-\frac{\Delta G^{\ddagger}}{RT}} \quad 6$$

where:
k is the reaction rate constant, k_B is the Boltzmann constant, T is the absolute temperature (in Kelvin), h is Planck's constant, $\Delta G^{\ddagger}$is the Gibbs free energy of activation, R is the universal gas constant.

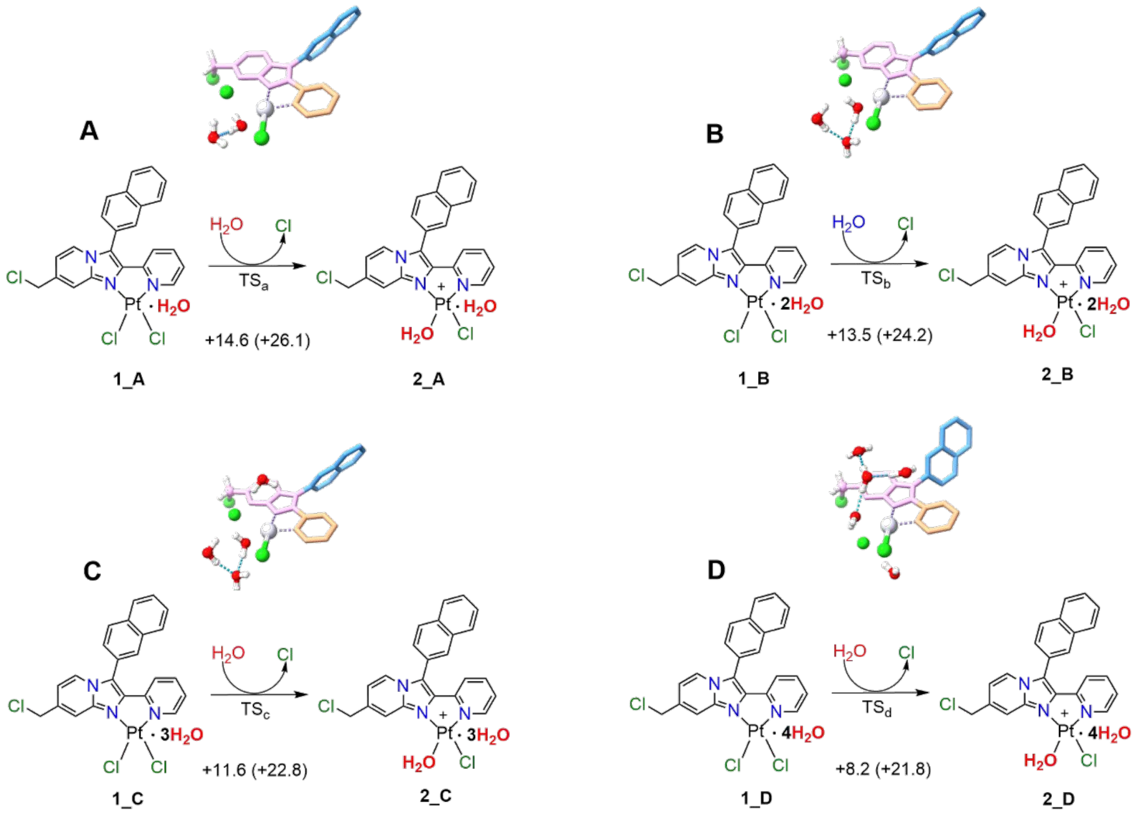

Figure S3. Activation energies (and Gibbs free energies in parenthesis) of the reaction between the Aurki-Pt compound and water considering microsolvation. All results were calculated at the B3LYP (PCM= water)/6-31+G**&LANL2DZ level of theory. Grimme's D3 and Baker & Johnson (BJ) dispersion-corrected methods were considered. Values are in kcalmol-1. Transition structures are on the top of the arrows.

Table S7 - Total electronic energies (E, in Hartree), zero-point vibrational energies (ZPVE, in Hartree), Thermal corrections to Gibbs Free Energies (TCGFE, in Hartree), and number of imaginary frequencies (NIMAG) of all the structures in Figure S3.

Structure	Energy	ZPCE	TCGFE	NIMAG(ν)
1_A	-2626.897600	0.363675	0.299705	0
2_A	-2242.950647	0.390059	0.327721	0
1_B	-2703.352539	0.388676	0.320759	0
2_B	-2319.399442	0.413969	0.345818	0
1_C	-2779.802352	0.412235	0.339350	0
2_C	-2395.857569	0.438963	0.370006	0
1_D	-2856.250524	0.436137	0.357482	0
2_D	-2472.309262	0.463232	0.388679	0
TS_a	-2703.319274	0.389261	0.324540	1(-136.5877)
TS_b	-2779.431550	0.413692	0.344135	1(-159.6085)
TS_c	-2856.228409	0.437239	0.363310	1(-155.4756)
TS_d	-2932.681998	0.461962	0.385295	1(-158.7426)

The bond-breaking/forming process from reactant (R) to product (P) via the cyclic transition structure TS was quantified by the calculation of the reaction synchronicity (sy) [4–8]. The synchronicity, calculated at B3LYP/6-31G*&LANL2DZ level of theory, is defined as

$$Sy = 1 - (2n-2)^{-1} \sum_{i=1}^{n} \frac{|\delta B_i - \delta B_{Av}|}{\delta B_{Av}} \qquad 7$$

where n is the number of bonds directly involved in the reaction, δB_i is the relative variation of a given bond index B_i at the TS as in the formula:

$$\delta B_i = \frac{|B_i^{TS} - B_i^{R}|}{|B_i^{P} - B_i^{TS}|} \qquad 8$$

where B_i is the bond order related to R, P and TS. The average value of δB_i denoted as δB_{Av} is

$$\delta B_{Av} = n^{-1} \sum_{i=1}^{n} \delta B_i. \qquad 9$$

Table S8. Properties calculated at DFT level of theory for Aurkine16 within different microsolvation environment.

**Microsolvation**	**0 H_2O**	**1 H_2O**	**2 H_2O**	**3 H_2O**	**4 H_2O**
Energy Orbital EHOMO (eV)	-6.22	-6.27	-6.28	-6.32	-6.30
Energy Orbital ELUMO (eV)	-2.55	-2.57	-2.57	-2.62	-2.62
Bond Distance O-Pt	2.96	2.89	2.87	2.90	2.90
Bond Distance Cl-Pt	2.77	2.45	2.48	2.47	2.47
Bond Angle (θ)	66.7	66.7	66.2	65.7	65.7
Energy Gap (ΔE)	3.68	3.70	3.71	3.71	3.67
Electronegativity (χ)	4.39	4.42	4.43	4.47	4.46
Absolute Hardiness (η)	1.84	1.85	1.85	1.85	1.84
Chemical Potential (π)	-4.39	-4.42	-4.43	-4.47	-4.46
Absolute softness (σ)	0.54	0.54	0.54	0.54	0.54
Electrophilicity (ω)	5.23	5.27	5.28	5.39	5.42
Relative variation of the bond index (δB_i)	0.51	0.53	0.50	0.49	0.49
Synchronicity (Sy)	0.84	0.85	0.84	0.90	0.98

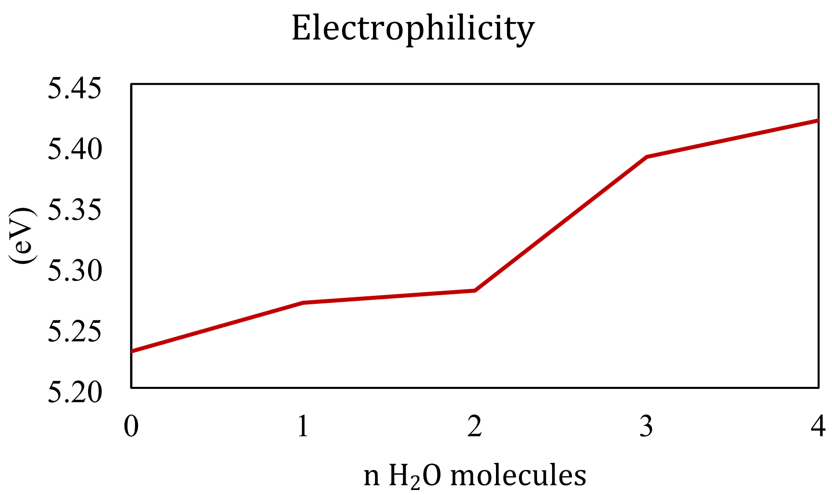

Figure S4. Variation of the electrophilicity with the quantity of explicit water molecules.

2. Reaction pathways analysis

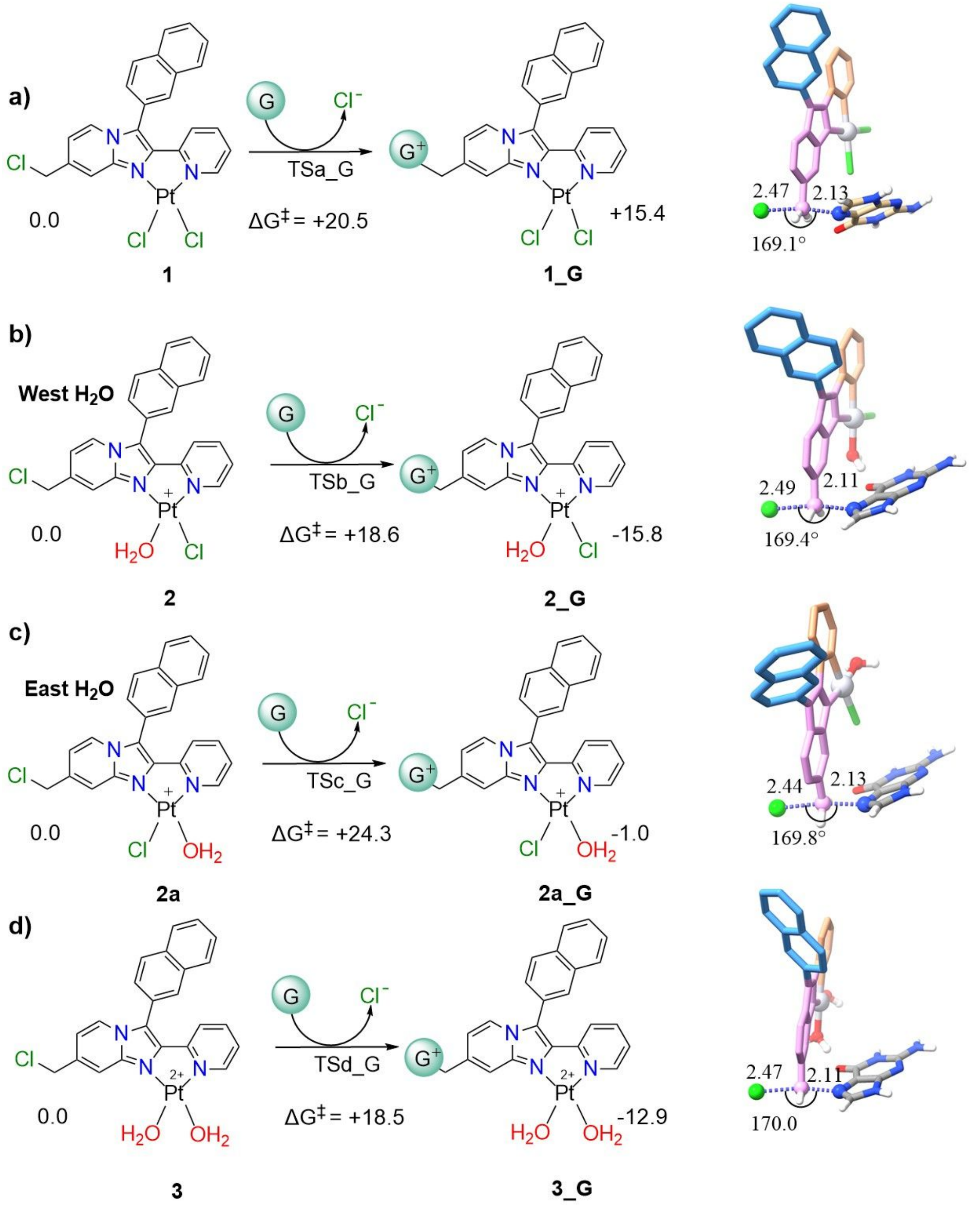

Figure S5. Reaction profile associated with the displacement of the Aurkine16's chloride, attached to the carbon, by a guanine molecule. Microsolvated environment was not considered. All results were calculated at the B3LYP (PCM= water)/6-31+G**&LANL2DZ level of theory. Grimme's D3 and Baker & Johnson (BJ) dispersion-corrected methods were considered. Values below structures or arrows correspond to relative or activation Gibbs energies, respectively (in kcal/mol) computed at 298.15 K.

Table S9 – Total electronic energies (E, in Hartree), zero-point vibrational energies (ZPVE, in Hartree), Thermal corrections to Gibbs Free Energies (TCGFE, in Hartree), and number of imaginary frequencies (NIMAG) of all the structures in Figure S5.

Structure	Energy	ZPCE	TCGFE	NIMAG(ν)
1	-2550.446145	0.340090	0.280715	0
1_G	-2632.730743	0.459848	0.391920	0
2	-2166.491382	0.365320	0.306217	0
2_G	-2248.772492	0.485522	0.418763	0
2a	-2166.491382	0.365320	0.306217	0
2a_G	-2248.774910	0.485141	0.417179	0
3	-1782.524223	0.390662	0.332188	0
3_G	-1864.805857	0.510851	0.444467	0
TSa_G	-3093.061733	0.456769	0.456769	1(-360.4545)
TSb_G	-2709.113430	0.482892	0.413061	1(-368.8344)
TSc_G	-2709.100384	0.481661	0.409116	1(-373.5518)
TSd_G	-2325.144608	0.507317	0.436796	1(-381.2603)

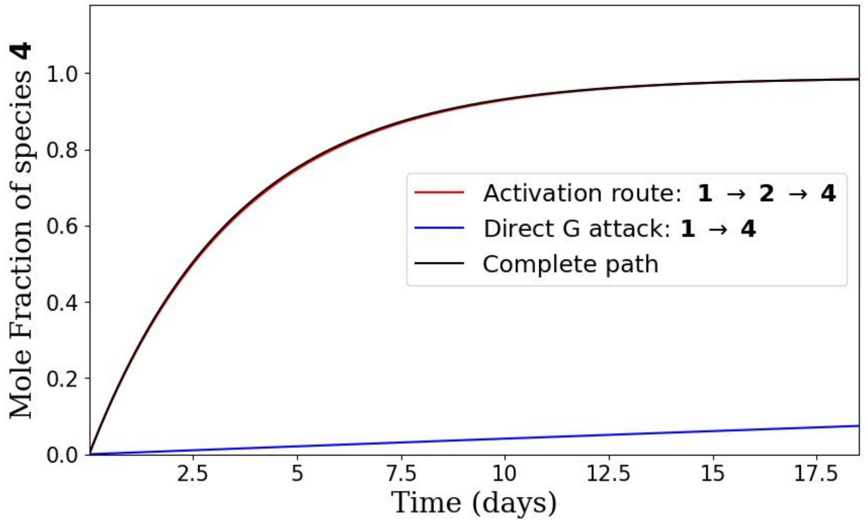

Figure S6. Simulated mole fraction of species 4 following the proposed activation route **1→2→4** (blue), the direct attack to guanine from the dichloride Aurkine16 **1→4** (red) and the complete path, considering both possibilities at once.

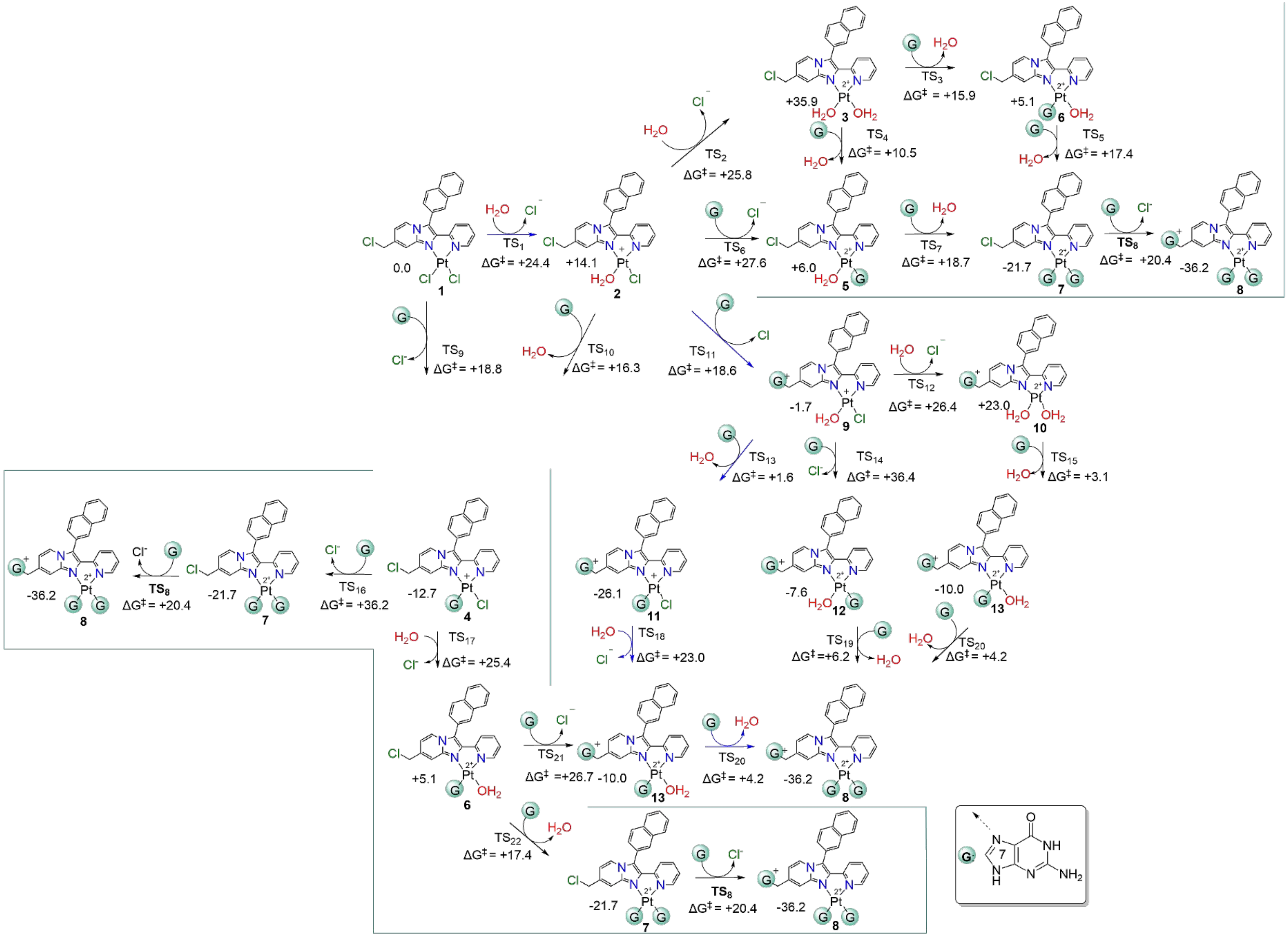

Figure S7. Map containing all the routes calculated in this work to reach the [**8**]$^{3+}$ product.

Table S10 - Total electronic energies (E, in Hartree), zero-point vibrational energies (ZPVE, in Hartree), Thermal corrections to Gibbs Free Energies (TCGFE, in Hartree), and number of imaginary frequencies (NIMAG) of all the structures in Figure S6.

Structure	Energy	ZPCE	TCGFE	NIMAG(ν)
H2O	-76.4419506	0.021215	0.021215	0
Cl^-	-460.381420	0.000000	-0.015023	0
Guanine	-542.628882	0.116637	0.083657	0
1	-2550.446145	0.340090	0.280715	0
2	-2166.490534	0.365175	0.305712	0
3	-1782.524223	0.390662	0.332188	0
4	-2632.723377	0.458024	0.388915	0
5	-2248.760795	0.483366	0.414256	0
6	-2248.763575	0.483649	0.415494	0
7	-2714.994533	0.576037	0.496973	0
8	-2797.393854	0.692629	0.607541	0
9	-2248.772492	0.485522	0.418763	0
10	-1864.805857	0.510851	0.444467	0
11	-2715.007942	0.579212	0.503268	0
12	-2331.041998	0.602929	0.524917	0
13	-2331.051811	0.604193	0.530967	0
TS_1	-2626.866931	0.364040	0.302027	1(-159.9805)
TS_2	-2242.909316	0.389356	0.327262	1(-162.9875)
TS_3	-3093.065991	0.457201	0.385289	1(-135.4087)
TS_4	-2709.1138827	0.481372	0.409899	1(-135.9326)
TS_5	-2709.096898	0.482333	0.410794	1(-124.4699)
TS_6	-2325.156575	0.507076	0.436110	1(-122.6704)
TS_7	-2325.146703	0.506521	0.434826	1(-123.9419)
TS_8	-2709.143215	0.482042	0.410839	1(-157.2519)
TS_9	-3175.317017	0.575894	0.495062	1(-113.4249)
TS_{10}	-2791.378768	0.598830	0.516864	1(-111.5693)
TS_{11}	-2791.386801	0.601012	0.521231	1(-118.7645)
TS_{12}	-3257.596981	0.693577	0.604950	1(-350.3096)
TS_{13}	-2709.113430	0.482892	0.413061	1(-368.8344)
TS_{14}	-2325.193368	0.508938	0.438344	1(-118.5960)
TS_{15}	-2791.426236	0.602267	0.524473	1(-108.9087)
TS_{16}	-2791.369717	0.602613	0.523738	1(-126.2969)
TS_{17}	-2407.449315	0.626978	0.547593	1(-137.5739)
TS_{18}	-2791.429692	0.601304	0.522755	1(-151.5366)
TS_{19}	-2791.373449	0.601140	0.522775	1(-357.5807)
TS_{20}	-2873.695156	0.721749	0.635727	1(-118.5271)

3. Molecular Dynamics Simulations

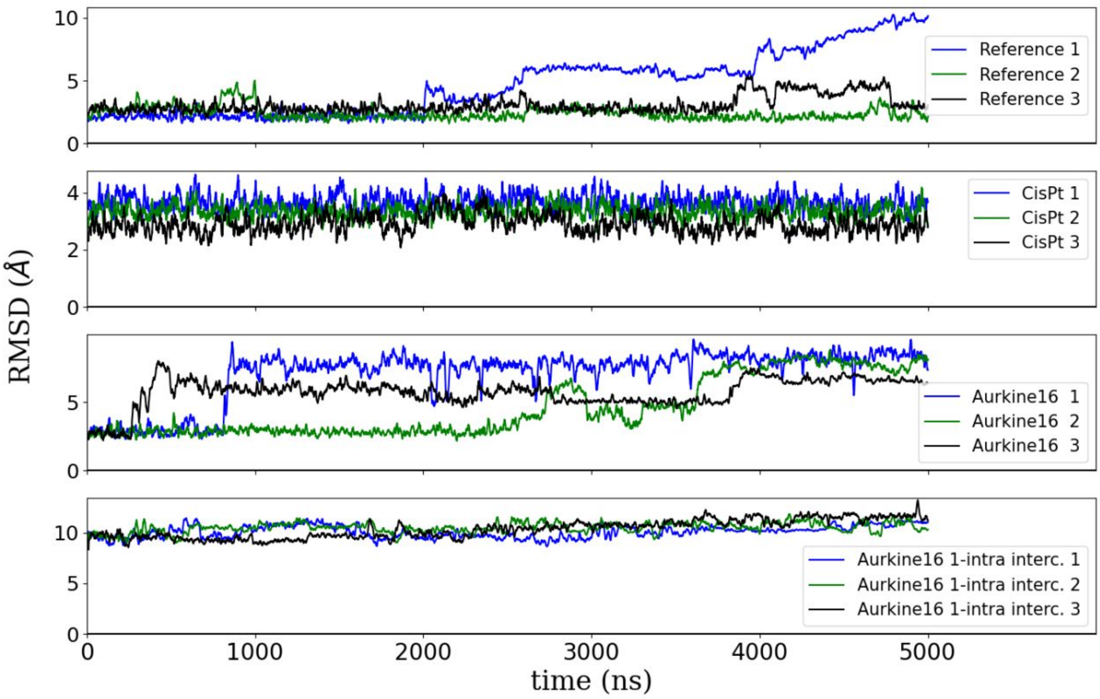

Figure S8. Root Mean Square Deviation of the DNA oligomer throughout the simulation time for the three runs performed (black, green, and blue lines, respectively), corresponding to the simulations described in the section *Aurkine16 Binding and Intercalation to an 18-mer B-DNA Sequence*. Specifically, these simulations include the DNA oligomer without cofactors (reference), the DNA oligomer in the presence of Cis-Pt(II) and Aurkine16 (Aurkine), and the specific case where Aurkine16 is covalently bonded and intercalated (1-intra). For ease the visualization, the running average of the RMSD is plotted considering the previous 10 frames.

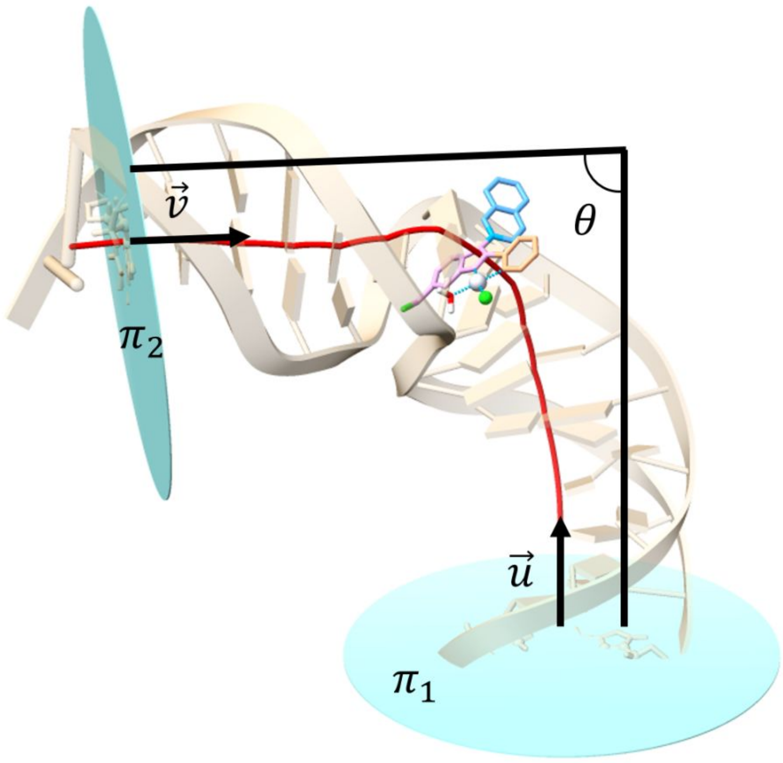

Figure S9. Illustration of the total bend angle calculation, defined as the angle formed by the vectors $\vec{u}$ and $\vec{v}$, which are normal to planes π1 and π2. For this calculation, the edge residues at the 5’ and 3’ ends were excluded. Further details on the definition of the planes can be found in reference [9].

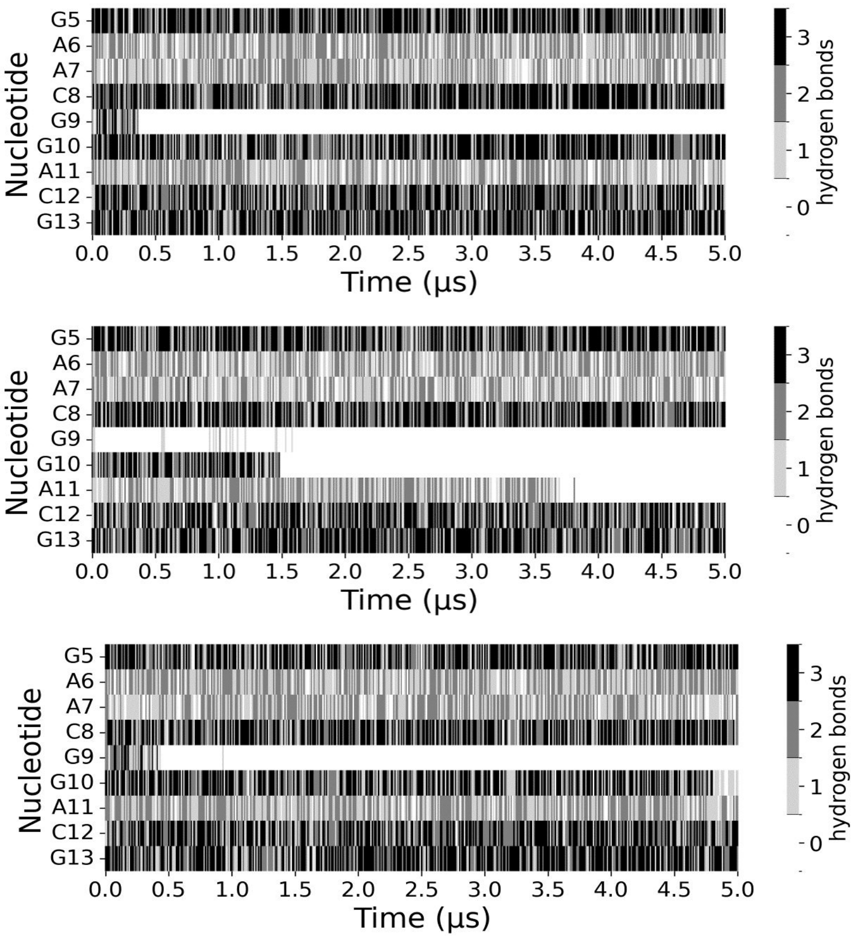

Figure S10. Hydrogen bond analysis for Aurkine16 intercalated with the 1-intra covalent bond across the three simulated replicas. The number of Watson-Crick hydrogen bonds was calculated throughout the simulation time. The CPPTRAJ code used for this analysis is provided below.

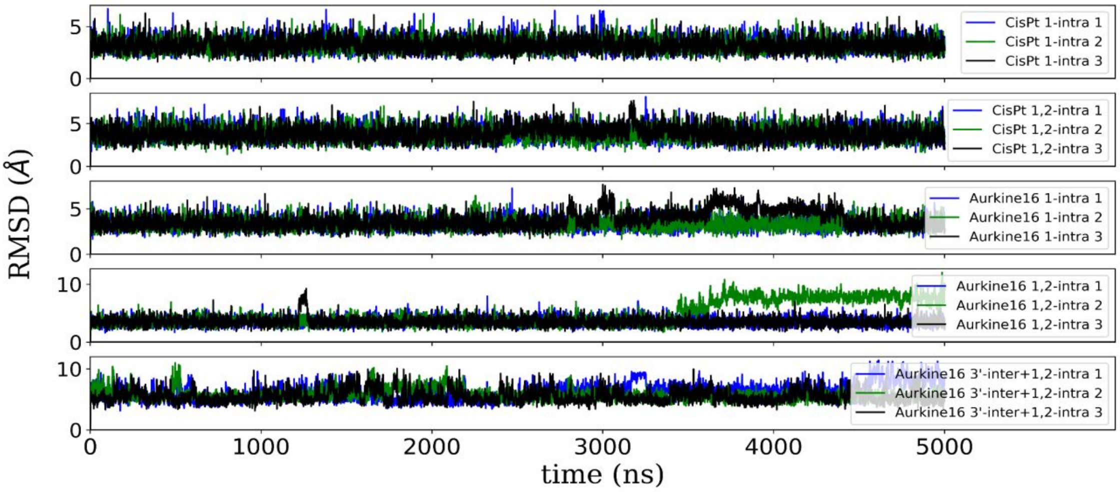

Figure S11. Root Mean Square Deviation of the DNA oligomer over the simulation time for the three performed runs (represented by black, green, and blue lines, respectively). This figure corresponds to the simulations described in the section *Aurkine16 binding via a CisPt-like mechanism*, which include Cisplatin with one and two covalent bonds (1-intra and 1,2-intra, respectively) and Aurkine16 with one, two, and three covalent bonds (1-intra, 1,2-intra, and 3'-inter+1,2-intra, respectively). A depiction of the simulated systems can be found in Figure 11A of the main text.

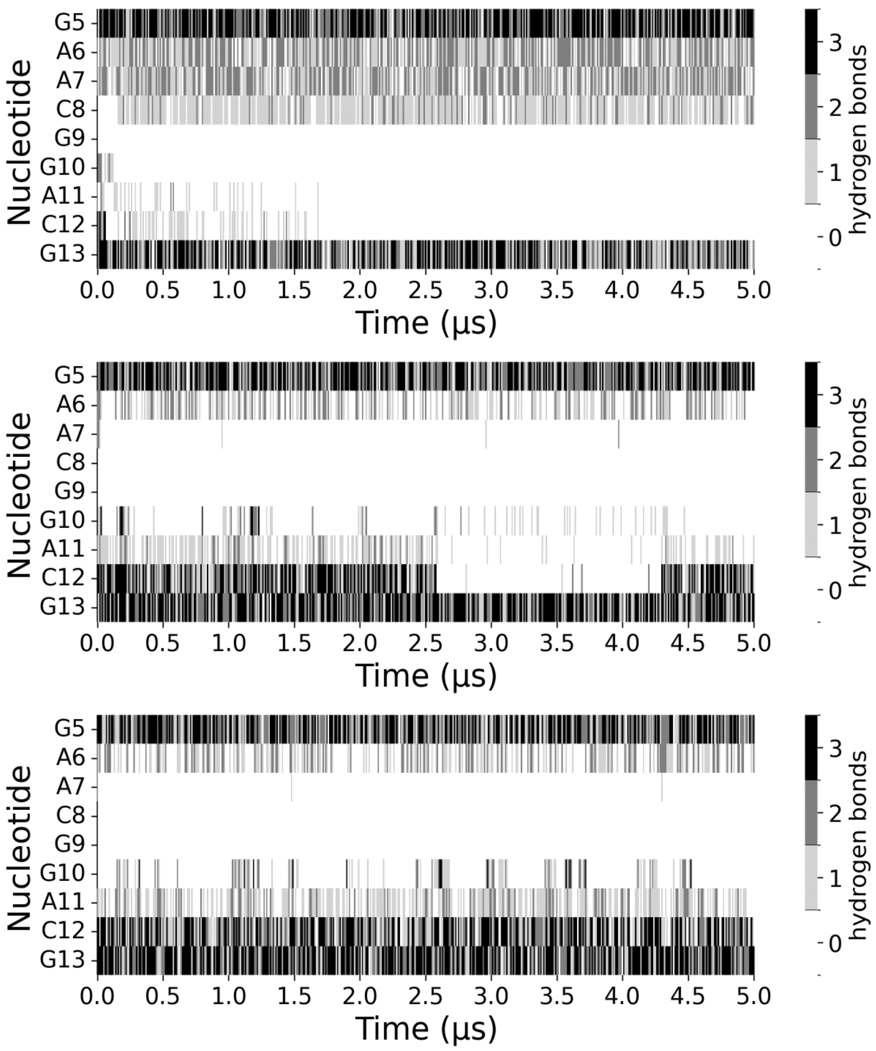

Figure S12. Hydrogen bond analysis for Aurkine16 3'-inter+1,2-intra bound to DNA across the three simulated replicas. The number of Watson-Crick hydrogen bonds was calculated throughout the simulation time. The CPPTRAJ code used for this analysis is provided below.

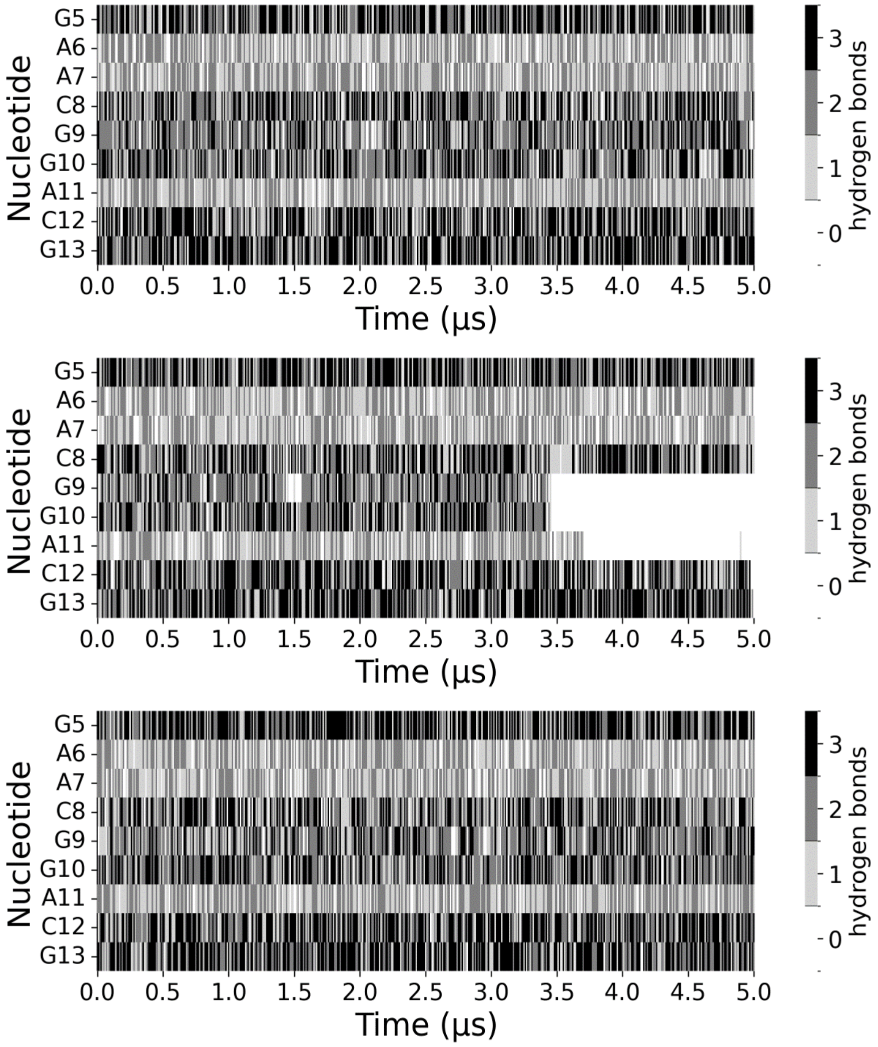

Figure S13. Hydrogen bond analysis for Aurkine16 1,2-intra bound to DNA across the three simulated replicas. The number of Watson-Crick hydrogen bonds was calculated throughout the simulation time. The CPPTRAJ code used for this analysis is provided below.

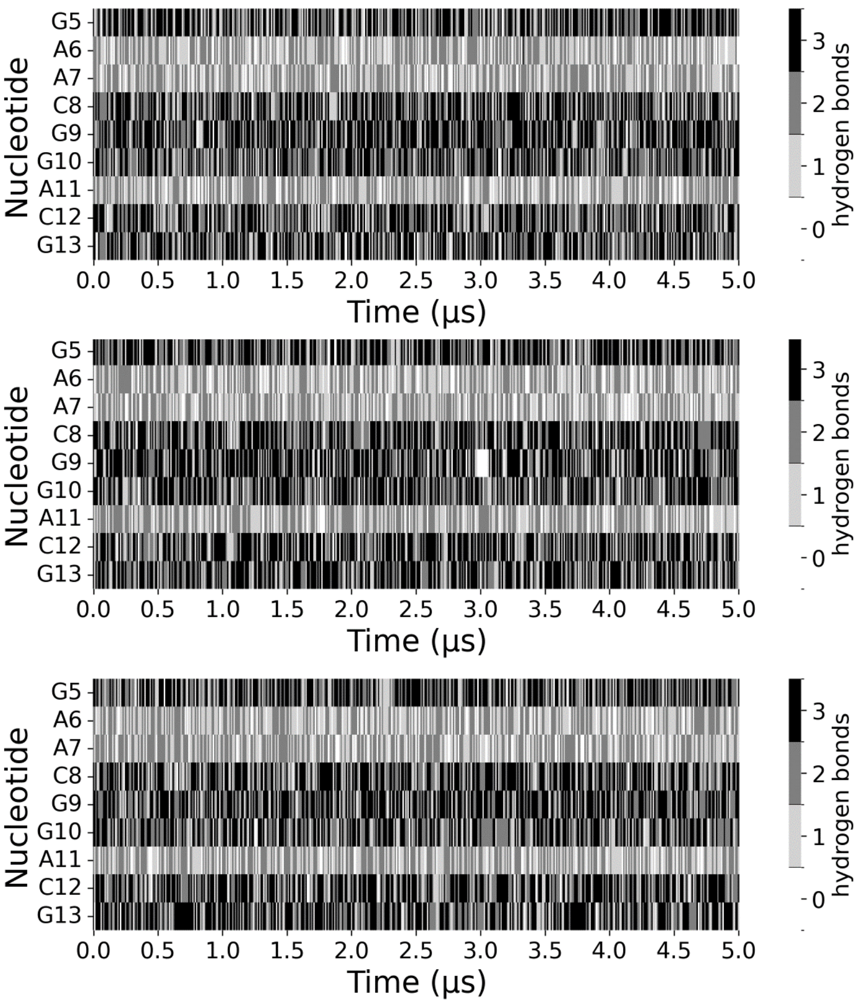

Figure S14. Hydrogen bond analysis for Aurkine16 1-intra bound to DNA across the three simulated replicas. The number of Watson-Crick hydrogen bonds was calculated throughout the simulation time. The CPPTRAJ code used for this analysis is provided below.

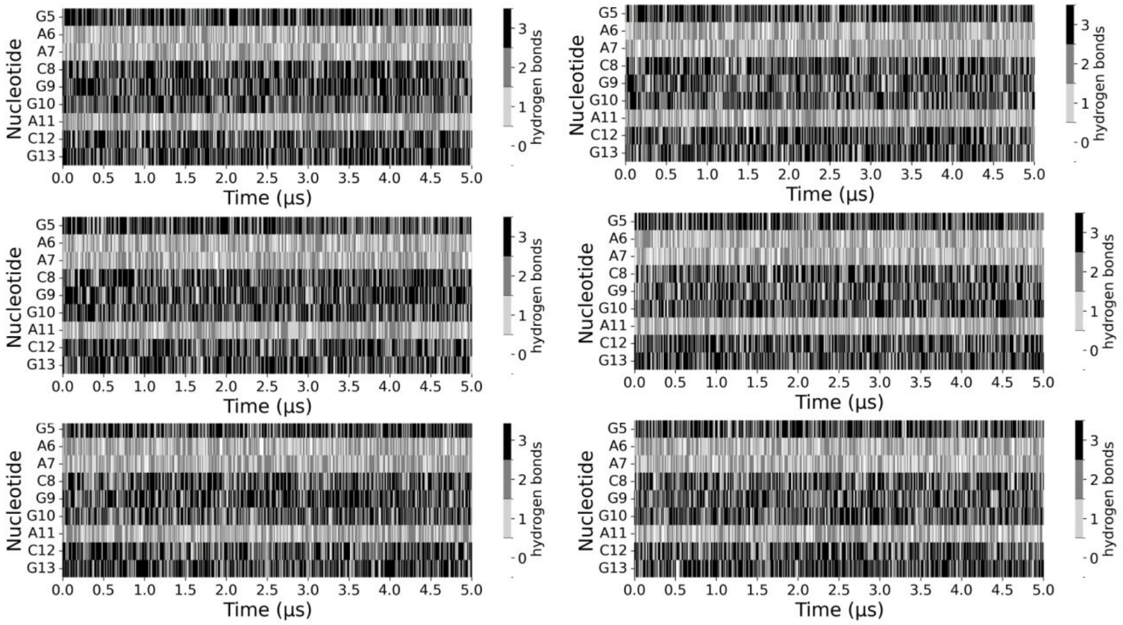

Figure S15. Hydrogen bond analysis for Cisplatin 1-intra bound to DNA (left) and Cisplatin 1,2-intra bound to DNA across the three simulated replicas. The number of Watson-Crick hydrogen bonds was calculated throughout the simulation time. The CPPTRAJ code used for this analysis is provided below.

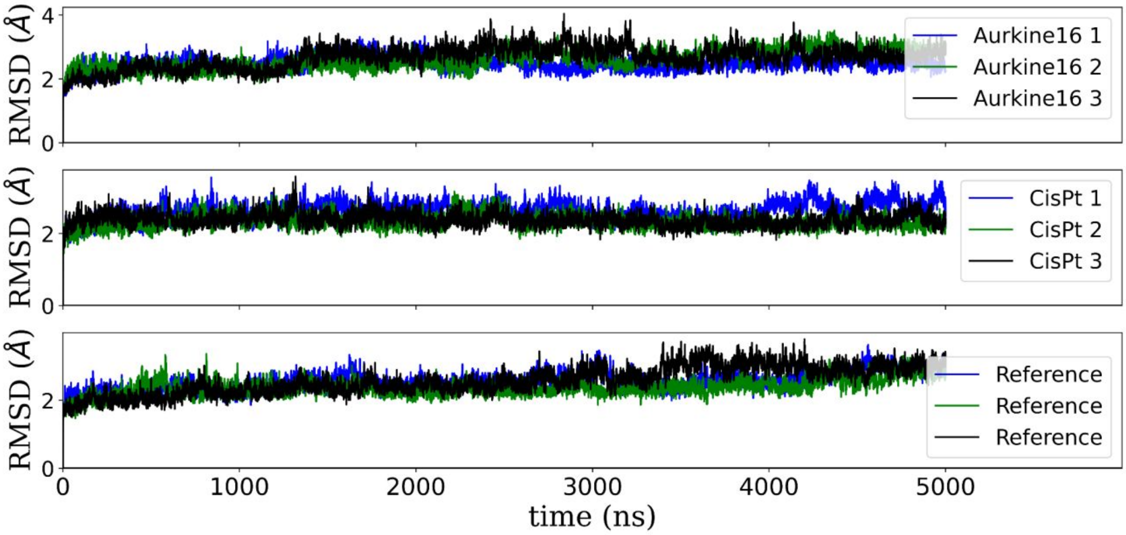

Figure S16. Root Mean Square Deviation of the DNA in the nucleosome core particle throughout the simulation time for the three runs performed (black, green, and blue lines, respectively). This relates to the simulations described in the section *"Aurkine16's Behaviour on the Nucleosome"*, which involve the nucleosome core particle (reference: PDB 1KX5) in the presence of four Aurkine16 molecules (Aurkine16) and four Cisplatin molecules (CisPt). Aurkine16 is depicted in Figure 12 of the main text.

Table S11 - Off-target contact analysis of Pt ions belonging to Cis-Pt(II) and Aurkine16. Each observed contact is described with the residue index in our system and the location based on the pdb-based convention of (PDB ID: 1KX566). For each contact, the percentage of frames in which at least one Pt ion is in contact (less than 4.5 Å) is provided together for both Cisplatin and Aurkine16 simulations, together with a comment on the specific location of the amino acid. The contact fractions have been computed with an in-house provided in the supporting information.

Residue	Location	Contact fraction Cisplatin (%)	Contact fraction Aurkine 16(%)	Index[b]
His-1252	H4-106	1.26	-	SE
His-765	H2B.2-106	1.03	-	SE
His-738	H2B.2-79	1.00	-	I
His-705	H2B.2-46	0.18	-	SE
His-333	H3-39	0.,14	-	T
His-934	H4-18	0.13	-	T
His-991	H4-75	-	0,08	SE
His-1100	H2A.2-82	0.03	-	I
His-1049	H2A.2-31	0.02	-	I
His-820	H3-39	0.01	0,02	T
Met-1202	H2B.2-56	0.83	-	I
Met-513	H4-84	0.56	-	I
Met-715	H2B.2-56	0.42	-	I
Met-901	H3-120	0.40	-	SE
Met-414	H3-120	0.37	-	SE
Met-384	H3-90	0.14	-	I
Met-871	H3-90	0.03	-	I

[b] SE =Solvent exposure, I= Internal , T= Tail

References

(1) Pearson, R. G. Absolute Electronegativity and Hardness Correlated with Molecular Orbital Theory. *Proceedings of the National Academy of Sciences* **1986**, *83* (22), 8440–8441.

(2) Parr, R. G.; Pearson, R. G. Absolute Hardness: Companion Parameter to Absolute Electronegativity. *J Am Chem Soc* **1983**, *105* (26), 7512–7516.

(3) Chattaraj, P. K.; Sarkar, U.; Roy, D. R. Electrophilicity Index. *Chem Rev* **2006**, *106* (6), 2065–2091.

(4) Lecea, B.; Arrieta, A.; Roa, G.; Ugalde, J. M.; Cossio, F. P. Catalytic and Solvent Effects on the Cycloaddition Reaction between Ketenes and Carbonyl Compounds To Form 2-Oxetanones. *J Am Chem Soc* **1994**, *116* (21), 9613–9619.

(5) Lecea, B.; Arrieta, A.; Lopez, X.; Ugalde, J. M.; Cossio, F. P. On the Stereochemical Outcome of the Catalyzed and Uncatalyzed Cycloaddition Reaction between Activated Ketenes and Aldehydes to Form Cis- and Trans-2-Oxetanones. An Ab Initio Study. *J Am Chem Soc* **1995**, *117* (49), 12314–12321.

(6) Moyano, A.; Pericas, M. A.; Valenti, E. A Theoretical Study on the Mechanism of the Thermal and the Acid-Catalyzed Decarboxylation of 2-Oxetanones (.Beta.-Lactones). *J Org Chem* **1989**, *54* (3), 573–582.

(7) Laurent, A. D.; Adamo, C.; Jacquemin, D. Dye Chemistry with Time-Dependent Density Functional Theory. *Phys. Chem. Chem. Phys.* **2014**, *16* (28), 14334–14356.

(8) Adamo, C.; Jacquemin, D. The Calculations of Excited-State Properties with Time-Dependent Density Functional Theory. *Chem. Soc. Rev.* **2013**, *42* (3), 845–856.

(9) Blanchet, C.; Pasi, M.; Zakrzewska, K.; Lavery, R. CURVES+ Web Server for Analyzing and Visualizing the Helical, Backbone and Groove Parameters of Nucleic Acid Structures. *Nucleic Acids Res* **2011**, *39* (suppl_2), W68–W73.
